# Data-informed insights into sex differences in peripheral blood mononuclear cells from single-cell transcriptomics

**DOI:** 10.1016/j.gendis.2025.101525

**Published:** 2025-01-08

**Authors:** Hui-Qi Qu, Joseph T. Glessner, Charlly Kao, Hakon Hakonarson

**Affiliations:** a The Center for Applied Genomics, Children's Hospital of Philadelphia, Philadelphia, PA 19104, USA; b Department of Pediatrics, The Perelman School of Medicine, University of Pennsylvania, Philadelphia, PA 19104, USA; c Division of Human Genetics, Children's Hospital of Philadelphia, Philadelphia, PA 19104, USA; d Division of Pulmonary Medicine, Children's Hospital of Philadelphia, Philadelphia, PA 19104, USA; e Faculty of Medicine, University of Iceland, Reykjavik 101, Iceland

This study employs data-driven approaches to analyze single-cell transcriptomic differences between male and female subjects in subpopulations of peripheral blood mononuclear cells (PBMCs). Applying a RandomForestRegressor, we observed consistent model performance across different samples. We reveal significant sex differences in PBMC subpopulations, most notably in four cell types: CD4^+^ Th2, CD8^+^ naïve T, CD4^+^ memory Treg, and CD4^+^ Th1/17 cells.

Sex differences in immune responses have attracted significant research attention, yet the underlying cellular mechanisms remain incompletely understood. Recent advancements in single-cell transcriptomics have provided unprecedented insights into the heterogeneity of immune cell populations. PBMCs are pivotal in immune research for their ability to reflect systemic immune responses and serve as biomarkers for disease states. Understanding how these cells vary between sexes at the molecular level could elucidate fundamental differences in immune function and susceptibility to diseases. Huang et al revealed differences in immune cell populations and gene expression between males and females, with implications for adaptive immune responses and inflammation.^1^ Our study builds on this by leveraging computational methods to rank cell types affected by sex, enabling quantitative comparisons and providing deeper insights into how specific PBMC subpopulations are influenced at the single-cell level. In this study, we hypothesize that there are significant sex differences in gene expression profiles across PBMC subpopulations, reflecting distinct cellular functions that contribute to variations in immune responses between males and females. We utilized a machine learning algorithm to analyze transcriptomic changes through single-cell analysis of PBMC subtypes from male and female subjects, aiming to uncover nuanced insights into sex-specific immune responses, paving the way for precision medicine and targeted immunotherapies. Weighted gene co-expression network analysis (WGCNA), in particular, allowed us to identify clusters of co-expressed genes, helping to elucidate gene networks that may play critical roles in these sex-based immune differences.

The single-cell RNA sequencing of PBMCs and data processing using the Seurat R package^2^ from nine independent pairs of de-identified female and male pediatric participants. The pairwise study design was matched for age, race, demographic information, sampling process, and single-cell RNA sequencing experiment conditions. A total of 15 cell types were classified using SingleR and the celldex:DatabaseImmuneCellExpression Data() function.^3^ To identify which cell types were most affected by sex differences, a RandomForestRegressor using scikit-learn^4^ was chosen due to its ability to handle high-dimensional data and provide feature importance metrics. The RandomForestRegressor model achieved five cross-validation (CV) mean squared error (MSE) scores (0.00573, 0.00710, 0.00577, 0.00584, and 0.00620), with a mean CV MSE of 0.00613 and a standard deviation of 0.00051. The test MSE of 0.00654 is close to the mean CV MSE, indicating consistent model performance. The 15 cell types along with their corresponding feature importance values are shown in Table 1. The four T cell types that account for the top 50% cumulative importance in the context of sex effects on gene expression in different cell types are: CD4^+^ Th2 cells, CD8^+^ naïve T cells, CD4^+^ memory Treg cells, and CD4^+^ Th1/17 cells. All four cell types have permutation *P*-values < 0.01. The permutation *P*-values indicate statistically significant importance rankings for 13 out of 15 cell types, highlighting the robustness of their contributions to the model and aligning with the known biological roles of these cell types in mediating sex-based immune responses.

**Table 1:** Cell types with corresponding feature importance values.

Cell type	Importance	Permutation *p*-value^a^
T cells, CD4^+^, Th2	0.2632	0.00
T cells, CD8^+^, naïve	0.1172	0.00
T cells, CD4^+^, memory Treg	0.0960	0.00
T cells, CD4^+^, Th1/17	0.0893	0.00
T cells, CD4^+^, Tfh	0.0547	0.00
T cells, CD4^+^, naïve	0.0526	0.00
T cells, CD4^+^, naïve Treg	0.0478	0.87
Monocytes, CD14^+^	0.0394	0.00
Natural killer cells	0.0389	0.00
B cells, naïve	0.0383	0.00
T cells, CD4^+^, Th17	0.0380	0.00
T cells, CD4^+^, naïve, stimulated	0.0351	1.00
T cells, CD8^+^, naïve, stimulated	0.0349	0.00
Monocytes, CD16^+^	0.0314	0.00
T cells, CD4^+^, Th1	0.0231	0.00

a Based on 100 permutations.

The RandomForestRegressor identified the most important PBMC cell types driving sex-specific gene expression differences, while WGCNA provided insights into the gene modules underlying these differences. Together, these methods offer a comprehensive understanding of how cell types and gene modules interact to mediate sex-specific immune responses. The WGCNA analysis identified several modules with differential expression between sexes, with effects that are cell type-specific (Fig. S2). For *CD4*^*+*^ Th2 cells, there was a weak negative correlation with the pink module (−0.124). *CD8*^*+*^ naïve T cells showed a weak positive correlation with the turquoise module (0.203). *CD4*^*+*^ memory Treg cells were negatively correlated with multiple modules, including the red module (−0.163), the pink module (−0.126), and the grey module (−0.131). *CD4*^*+*^ Th1/17 cells exhibited a weak positive correlation with the grey module (0.196). A more detailed discussion is provided in the *Supplementary Information*. Genes within each module, hub genes, and over-representation analysis are detailed in *Supplementary Information* and Table S2.

Our study found that sex significantly could influence the transcriptome of various PBMC cell types. Notably, four cell types showed the most sex-specific differences, including *CD4*^*+*^ Th2 cells, *CD8*^*+*^ naïve T cells, *CD4*^*+*^ memory Treg cells, and *CD4*^*+*^ Th1/17 cells, accounting for over 50% of the sex effects. *CD4*^*+*^ Th2 cells play a crucial role in orchestrating immune responses by producing cytokines, such as IL-4, IL-5, and IL-13. *CD8*^*+*^ naïve T cells are critical for initiating cytotoxic responses against intracellular pathogens and tumors. The significant sex-specific differences observed in *CD8*^*+*^ naïve T cells could illuminate the differential susceptibility and immune response to viral infections and cancers between males and females. *CD4*^*+*^ memory Treg cells are essential for maintaining immune tolerance and preventing autoimmunity. Th1/17 cells are implicated in various autoimmune and chronic inflammatory conditions. Less efficient control of autoimmune Th1/17 cell responses has been observed in female rats compared with males. The sex differences in *CD4*^*+*^ memory Treg and Th1/17 cells observed in our study may help explain the higher prevalence of autoimmune diseases in females.

In contrast to the above four cell types, the cell types less affected by sex differences often play fundamental roles in both innate and adaptive immunity, *e.g.*, monocytes, natural killer cells, and naïve B cells. These roles are critical for immediate and effective responses to infectious agents, which have exerted strong evolutionary pressure on the immune system.^5^ Ensuring robust and consistent functionality across sexes for these cell types could be vital for survival.

These findings have important clinical implications. The sex-specific differences in PBMC subtypes highlight the need for precision medicine approaches that consider sex as a biological variable. Tailored therapies targeting these cells could improve outcomes in autoimmune diseases, cancers, and infections by addressing distinct immune functions influenced by sex. For example, cytokine therapies or immune modulators may benefit from sex-based adjustments to optimize efficacy. In contrast, the consistent functionality of those cell types across sexes makes them robust targets for generalized therapies, such as monoclonal antibodies, natural killer cell activators, or B cell modulators, offering broad utility without requiring sex-specific modifications. However, while natural killer cells and monocytes exhibit minimal sex-specific differences, these differences are still statistically significant. This underscores that even small sex-based effects can subtly but meaningfully influence the regulation of immune responses. Further investigation into these subtypes could reveal how selective pressures, such as pathogen-mediated selection or reproductive immunological demands, have shaped these sex-specific differences. Understanding the mechanisms driving these variations could illuminate both sex-specific nuances and the conserved immune functions critical for survival.

Taken together, the identification of sex-specific differences in the transcriptomes of these key PBMC cell types underscores the importance of considering sex as a biological variable in immunological research. These differences provide insights into the mechanisms driving sex-based disparities in immune responses, susceptibility to infections, autoimmune diseases, and overall immune regulation. Further research into the cellular and molecular underpinnings of these differences could lead to more precise and effective therapeutic strategies accounting for sex-specific immune responses.

## Ethics declaration

All experimental protocols were approved by the Institutional Review Board (IRB) of the Children's Hospital of Philadelphia (CHOP) with the IRB number: IRB 16-013278. Informed consent was obtained from all subjects. If subjects were under 18, consent was also obtained from a parent and/or legal guardian with assent from the child if 7 years or older.

## Funding

The study was supported by the Institutional Development Funds from the 10.13039/100006458Children's Hospital of Philadelphia to the Center for Applied Genomics (USA) and The Children's Hospital of Philadelphia Endowed Chair in Genomic Research (USA) to Hakon Hakonarson.

## CRediT authorship contribution statement

**Hui-Qi Qu:** Writing – review & editing, Writing – original draft, Methodology, Investigation, Formal analysis, Conceptualization. **Joseph T. Glessner:** Resources. **Charlly Kao:** Project administration. **Hakon Hakonarson:** Writing – review & editing, Supervision, Project administration, Funding acquisition, Conceptualization.

## Conflict of interests

The authors declared no potential conflict of interests with respect to the research, authorship, and/or publication of this article.
