# Supplementary material for: Data-informed insights into sex differences in peripheral blood mononuclear cells from single-cell transcriptomics
Source: Genes Dis. 2025 Jan 8;12(5):101525. doi: 10.1016/j.gendis.2025.101525 (PMC12164016; doi:10.1016/j.gendis.2025.101525)
Supplement: Multimedia component 1 [file mmc1.docx]

**Supplementary materials**

**Data-Informed Insights into Sex Differences in Peripheral Blood Mononuclear Cells from Single-Cell Transcriptomics**

Hui-Qi Qu^1^, Joseph T Glessner^1,2,3^, Charlly Kao^1^, Hakon Hakonarson^1,2,3,4,5*^.

**Supplementary Methods**

***PBMC scRNA-seq***

The scRNA-seq analysis of PBMCs and data processing using the Seurat R package^1;2^ from 9 independent de-identified male and female child pairs were previously detailed in our prior publication^3^. No subject has blood system malignancies, which can lead to dysregulation of blood cell proliferation and function. Each pair was considered an independent experiment. To minimize experimental bias, each male-female pair was processed in the same batch experiment. Additionally, we tested for potential batch effects by evaluating the distribution of principal components and clustering patterns across batches using Seurat’s integration diagnostics. No significant batch effects were observed. A total of 15 cell types were classified using SingleR and the celldex::DatabaseImmuneCellExpression Data() function^4^ for further analysis. 15 cell types classified by singleR and the DatabaseImmuneCellExpressionData function in the celldex R package^4^ were analyzed, including (counts and percentages per sample): T cells, CD4^+^, naive (1442, 22.6%); Monocytes, CD14^+^ (1362, 21.3%); B cells, naive (1204, 18.9%); NK cells (860, 13.5%); T cells, CD8^+^, naive (527, 8.2%); Monocytes, CD16^+^ (336, 5.3%); T cells, CD4^+^, naive TREG (111, 1.7%); T cells, CD4^+^, naive, stimulated (102, 1.6%); T cells, CD4^+^, Th2 (100, 1.6%); T cells, CD4^+^, Th1_17 (72, 1.1%); T cells, CD8^+^, naive, stimulated (72, 1.1%); T cells, CD4^+^, TFH (66, 1.0%); T cells, CD4^+^, Th1 (48, 0.7%); T cells, CD4^+^, Th17 (48, 0.7%); and T cells, CD4^+^, memory TREG (36, 0.6%).

The data were organized into a matrix where rows represented individual genes, and columns represented different cell types and subject pairs. Each entry in the matrix corresponded to the log2 fold change (log2FC) of female/male in gene expression for each cell type. Missing values for a specific gene per cell type in an experiment, due to insufficient cell numbers, were imputed by the mean log2FC from other experiments. Genes missing in certain cell types due to lack of expression were assigned a value of 0.

***Machine Learning***

To determine which cell types were most impacted by sex differences, we utilized a RandomForestRegressor from scikit-learn^5^. This method was selected for its capability to manage high-dimensional datasets and generate feature importance metrics. The dataset was split into training and testing sets, with 7 experiments for training and 2 for testing. K-fold cross-validation was applied within the training set to further divide it into training and validation subsets, ensuring robust model evaluation and parameter tuning. Different parameters and train-test split ratios were tested for the RandomForestRegressor (Supplementary Table 1, Supplementary Figure 1). Except for max_depth=10, which had poorer performance and could prevent the model from capturing complex relationships in the data, the results were highly consistent across different parameters and split ratios. For the final analysis, the number of trees (n_estimators) was set to 100, max_depth to none, min_samples_split to 2, and random_state to 42. Multiple train-test splits (60:40, 70:30, 80:20, and 90:10) were tested to evaluate the model's sensitivity to variations in dataset division. Splits with larger test sets, such as 60:40, resulted in higher CV MSEs due to reduced training data, which limited the model's ability to learn effectively. Smaller test sets, such as 90:10, resulted in lower CV MSEs but higher test MSEs, suggesting overfitting and reduced generalization to unseen data. Choosing the 0.8-0.2 train-test split is justified because it offers a balanced approach, providing sufficient data for both training and testing, leading to a reliable and generalizable model.

A 5-fold cross-validation (CV) was performed on the training set to assess the model's robustness and to avoid overfitting. The mean squared error (MSE) was calculated for each fold to evaluate model performance. After training, the feature importance values were extracted from the RandomForestRegressor. These importance values indicate the relative contribution of each cell type to the model's predictions. The cell types were ranked based on their importance scores. The permutation_importance function from sklearn.inspection is used to estimate the distribution of feature importance values by shuffling the feature values. 100 permutations for each feature were performed to estimate their statistical significance using permutation importance. For each feature, the p-value is calculated as the proportion of permuted importance values that are greater than or equal to the original values.

***WGCNA Analysis***

The WGCNA analysis was performed on 2649 DE genes with sex effects, out of which 2,626 genes were consistently detected across all samples. The average gene expression per cell type for each sample was computed using log-normalized values with the Seurat R package^1;2^. For the gene co-expression network construction, we utilized the WGCNA R package^6;7^. Connectivity was assessed across various soft-thresholding powers, revealing an improvement in the scale-free topology fit index (SFT.R^2) as power increased, with a peak value of 0.957 at power 8, indicating a strong scale-free network structure (Supplementary Figure 3a). The mean connectivity values decreased with increasing power, leading to a sparser network (Supplementary Figure 3b). Powers 6 and 7 were noted as particularly effective, with high SFT.R^2 values, suggesting their suitability for constructing a robust gene co-expression network. Power 7 was selected because it achieved an optimal trade-off, with connectivity values that supported meaningful module detection while maintaining a strong scale-free network structure. This power provided a balance between network complexity and module resolution, ensuring that the resulting modules were biologically interpretable and neither too coarse nor overly fragmented. Overrepresentation analysis (ORA) of Hallmark gene sets was conducted using the clusterProfiler R package^8^ and the msigdbr R package^9^.

**Supplementary Information**

***Due to space limitations, the following text is described in the main text, with references cited here.***

Sex differences in immune responses have attracted significant research attention^10^. Peripheral blood mononuclear cells (PBMCs) are pivotal in immune research for their ability to reflect systemic immune responses and serve as biomarkers for disease states^11^.

CD4^+^ Th2 cells play a crucial role in orchestrating immune responses by producing cytokines, such as IL-4, IL-5, and IL-13^12^. CD8^+^ naïve T cells are critical for initiating cytotoxic responses against intracellular pathogens and tumors^13^. The significant sex-specific differences observed in CD8^+^ naïve T cells could illuminate the differential susceptibility and immune response to viral infections and cancers between males and females. CD4^+^ memory Treg cells are essential for maintaining immune tolerance and preventing autoimmunity^14^. Th1/17 cells are implicated in various autoimmune and chronic inflammatory conditions^15^. Less efficient control of autoimmune Th1/17 cell responses have been observed in female rats compared to males^16^. The sex differences in CD4^+^ memory Treg and Th1/17 cells observed in our study may help explain the higher prevalence of autoimmune diseases in females.

***Extended discussion on cell type-specific module correlations***

To validate the biological relevance of the feature importance rankings, we cross-referenced the four top-ranked cell types with existing literature. The high importance of CD4^+^ Th2 cells aligns with their established role in sex-biased cytokine production, including IL-4, IL-5, and IL-13^17^. CD8^+^ naïve T cells are well-documented for their involvement in cytotoxic immune responses, which exhibit sex-specific differences, particularly in viral immunity and cancer susceptibility^18^. CD4^+^ memory Treg cells play a critical role in maintaining immune tolerance, and their differential activity between sexes has been linked to variations in autoimmune disease prevalence^17^. Similarly, the prominence of CD4^+^ Th1/17 cells is consistent with their known involvement in chronic inflammation and autoimmunity, which are more prevalent in females^19^. These findings confirm that the identified cell types have biologically plausible roles in sex-specific immune responses. The biological significance of the feature importance rankings is further supported by detailed analyses of module correlations within the four top-ranked cell types.

***CD4^+^ Th2 cells***

Females typically exhibit more robust humoral and Th2-mediated immune responses compared to males, which could reflect estrogen influences^10^. Our findings of significant sex differences in Th2 cells could have profound implications for understanding sex-based disparities in immune responses and allergic reactions, where Th2 responses are key.

Interestingly, while the pink module is significantly upregulated in females and in several cell types (e.g., naïve B cells, CD14^+^ Monocytes, CD16^+^ Monocytes), CD4^+^ Th2 cells show a negative correlation with the pink module, indicating lower expression levels. The Hub gene for the pink module, *SIPA1L1* (signal induced proliferation associated 1 like 1), is involved in the regulation of cytoskeletal dynamics through its interaction with the Rap1 GTPase pathway^20^. In CD4^+^ Th2 cells, proper cytoskeletal rearrangement is crucial for processes such as migration, immune synapse formation, and activation^21^. It is worth noting that the higher expression of *SIPA1L1* in females, despite its otherwise lower expression in CD4^+^ Th2 cells, could suggest that it may be more efficient to upregulate genes by sex that are typically expressed at lower levels in these cells, reflecting physiological differences in females.

***CD8^+^ naïve T cells***

While the turquoise module is significantly downregulated in females and in several cell types in females (e.g., CD16^+^ Monocytes, CD4^+^ naïve stimulated T cells, CD14^+^ Monocytes), CD8^+^ naïve T cells show a positive correlation with the turquoise module, indicating higher expression levels. The hub gene of the turquoise module, *RPL13* (Ribosomal Protein L13), encodes a component of the 60S subunit of the ribosome, playing a key role in protein synthesis^22^. In CD8^+^ naïve T cells, *RPL13* is essential for maintaining cellular homeostasis and supporting the high protein production required for their rapid proliferation and differentiation into cytotoxic T cells upon antigen encounter^23;24^. Proper ribosomal function, driven by *RPL13*, ensures that these cells can efficiently produce the proteins necessary for their activation and subsequent immune response.

The turquoise module is enriched for the HALLMARK_MYC_TARGETS_V1 and HALLMARK_OXIDATIVE_PHOSPHORYLATION gene sets. HALLMARK_MYC_TARGETS_V1 includes targets of the MYC transcription factor, which plays a pivotal role in regulating cell growth, metabolism, and proliferation^25^. In CD8^+^ naïve T cells, MYC activity is essential for rapid cell division and differentiation upon activation^26^. MYC drives the expression of genes involved in ribosome biogenesis, nucleotide synthesis, and metabolic reprogramming, all of which are crucial for the transition from a naïve state to an active cytotoxic T cell ready to respond to pathogens^27^. HALLMARK_OXIDATIVE_PHOSPHORYLATION includes components of the mitochondrial oxidative phosphorylation pathway, which is responsible for ATP production through the electron transport chain^28^. In CD8^+^ naïve T cells, efficient oxidative phosphorylation is necessary to meet the energy demands during activation and proliferation^29^. As these cells transition from a quiescent state to an active one, a shift towards increased oxidative phosphorylation supports the high energy requirements needed for rapid expansion and effective immune responses.

It is worth noting that the lower expression of the turquoise module in females, in contrast to its higher expression in CD4^+^ Th2 cells, could suggest that downregulating genes by sex that are typically expressed at higher levels in these cells may be more efficient, reflecting physiological differences in females.

***CD4^+^ memory Treg cells***

Estrogens and progesterone are known to reduce Treg cell development and function^30^. The sex differences in CD4^+^ memory Treg cells observed in our study may help explain the higher prevalence of autoimmune diseases in females.

In our analysis, CD4^+^ memory Treg cells showed a negative correlation with several modules, including the red, pink, and grey modules, indicating lower expression levels of these genes. In contrast, these modules have positive correlation with females. Despite their low baseline expression in CD4^+^ memory Treg cells, the hub genes for these modules, *EIF5* (red), *SIPA1L1* (pink), and *PIP4K2A* (grey), are likely important regulators in CD4^+^ memory Treg cells influenced by sex. *EIF5* (eukaryotic translation initiation factor 5) encodes a eukaryotic translation initiation factor, may be crucial in modulating protein synthesis and maintaining cellular homeostasis under various conditions^31^. *SIPA1L1* could influence cell migration, immune synapse formation, and other processes essential for the regulatory functions of Treg cells by the regulation of cytoskeletal dynamics^20^. *PIP4K2A* (phosphatidylinositol-5-phosphate 4-kinase type 2 alpha ) is likely involved in lipid signaling pathways that control various aspects of cell growth, survival, and immune response regulation^32^. Together, these genes may orchestrate a complex network of pathways that are vital for the proper functioning and stability of CD4^+^ memory Treg cells.

In addition, four HALLMARK gene sets are overrepresented in the grey module. In CD4^+^ memory Treg cells, the baseline expression of the HALLMARK_INTERFERON_GAMMA_RESPONSE, HALLMARK_INTERFERON_ALPHA_RESPONSE, HALLMARK_ESTROGEN_RESPONSE_EARLY, and HALLMARK_ESTROGEN_RESPONSE_LATE gene sets is generally low, reflecting the suppressive and regulatory functions of these cells in maintaining immune tolerance and preventing autoimmunity^33^. However, in females, these gene sets are upregulated, likely driven by estrogen and its signaling pathways, which enhance both interferon and estrogen responses^30^. Consistent with other cell types, the higher expression of these modules and gene sets in females, compared to their lower baseline expression in CD4^+^ memory Treg cells, may suggest that upregulating genes typically expressed at lower levels in these cells is more efficient, reflecting physiological differences between the sexes.

The HALLMARK_INTERFERON_ALPHA_RESPONSE and HALLMARK_INTERFERON_GAMMA_RESPONSE gene sets are particularly relevant in CD4⁺ memory Treg cells due to their roles in modulating the immune microenvironment during inflammation or infection^34^. Interferon-alpha (IFN-α) signaling has been shown to influence the function of Tregs by modulating their suppressive capacity^35^. Interferon-gamma (IFN-γ) responses are critical for Treg-mediated crosstalk with effector T cells^36^. The estrogen-related gene sets are also significant in CD4⁺ memory Treg cells, likely reflecting the interplay between estrogen and the regulatory role of Tregs in preventing autoimmune responses^37^.

***CD4^+^ Th1/17 cells***

In our study, we observed a positive correlation between the grey module and Th1/17 cells, with this module also upregulated in females. The unique response in Th1/17 cells, where females upregulate a gene set that is already highly expressed, differs from the pattern observed in other cell types, where upregulation typically occurs in gene sets with lower baseline expression. This difference may be attributed to the dual role of Th1/17 cells^15^, placing them at a crucial point in immune regulation. The upregulation of highly expressed gene sets in females may indicate heightened immune vigilance, potentially driven by estrogen, which enhances both interferon and estrogen signaling.

As a limitation of this study, log-normalization for the WGCNA analysis may introduce scaling biases that compress variability in highly expressed genes and exaggerate differences in lowly expressed genes. These biases could subtly affect the interpretation of module correlations, such as the weak associations observed in CD4^+^ Th2 cells with the pink module. For example, highly expressed hub genes like *SIPA1L1* in CD4^+^ Th2 cells may have their biological signals underestimated, while sex differences in modules correlated with lowly expressed genes, such as those in CD4^+^ memory Treg and Th1/17 cells, might be overemphasized. While these potential biases should be acknowledged, log-normalization is a well-established and robust method commonly used in RNA-seq studies. The consistent patterns observed across cell types and modules, as well as the biological plausibility of the identified correlations (e.g., roles of *SIPA1L1*), suggest that the overall conclusions of this study are unlikely to be significantly compromised. These findings align with established biological functions and literature, indicating that any biases introduced by log-normalization are unlikely to invalidate the key results but should be considered when interpreting weaker correlations or sex-specific differences.

**Supplementary Table 1 Different parameters and train-test split ratios tested for the RandomForestRegressor**

| **Condition** | **Cross-Validation MSE Scores** | **Mean CV MSE** | **Standard Deviation of CV MSE** | **Test MSE** |
| --- | --- | --- | --- | --- |
| a. n_estimators=200, max_depth=None, min_samples_split=2 | [5.70e-03, 7.06e-03, 5.71e-03, 5.74e-03, 6.08e-03] | 6.06E-03 | 5.23E-04 | 6.44E-03 |
| b. n_estimators=100, max_depth=10, min_samples_split=2 | [2.70e-02, 2.88e-02, 2.69e-02, 2.67e-02, 2.71e-02] | 2.73E-02 | 7.82E-04 | 2.79E-02 |
| c. n_estimators=100, max_depth=None, min_samples_split=5 | [5.86e-03, 7.23e-03, 5.84e-03, 5.94e-03, 6.39e-03] | 6.25E-03 | 5.31E-04 | 6.63E-03 |
| d. n_estimators=100, max_depth=None, min_samples_split=2 | [5.73e-03, 7.10e-03, 5.77e-03, 5.84e-03, 6.20e-03] | 6.13E-03 | 5.12E-04 | 6.54E-03 |
| e. Train-Test Split=60:40 | [7.40e-03, 6.55e-03, 6.39e-03, 6.59e-03, 6.64e-03] | 6.71E-03 | 3.53E-04 | 6.75E-03 |
| f. Train-Test Split=70:30 | [7.53e-03, 6.56e-03, 5.70e-03, 6.43e-03, 6.76e-03] | 6.60E-03 | 5.87E-04 | 6.40E-03 |
| g. Train-Test Split=80:20 | [5.73e-03, 7.10e-03, 5.77e-03, 5.84e-03, 6.20e-03] | 6.13E-03 | 5.12E-04 | 6.54E-03 |
| h. Train-Test Split=90:10 | [5.69e-03, 6.45e-03, 6.09e-03, 5.58e-03, 6.26e-03] | 6.01E-03 | 3.29E-04 | 6.91E-03 |

a-d: Train-Test Split=80:20; e-h: n_estimators=100, max_depth=None, min_samples_split=2

**Supplementary Table 2 Overrepresentation analysis of Hallmark gene sets in WGCNA modules**

| **Description** | **GeneRatio** | **BgRatio** | **pvalue** | **p.adjust** | **qvalue** | **Count** | **Module** |
| --- | --- | --- | --- | --- | --- | --- | --- |
| HALLMARK_INTERFERON_GAMMA_RESPONSE | 7/25 | 200/4383 | 8.84E-05 | 2.21E-03 | 1.77E-03 | 7 | grey |
| HALLMARK_INTERFERON_ALPHA_RESPONSE | 5/25 | 97/4383 | 1.79E-04 | 2.24E-03 | 1.79E-03 | 5 | grey |
| HALLMARK_ESTROGEN_RESPONSE_EARLY | 6/25 | 200/4383 | 7.16E-04 | 5.96E-03 | 4.77E-03 | 6 | grey |
| HALLMARK_ESTROGEN_RESPONSE_LATE | 5/25 | 200/4383 | 4.73E-03 | 2.96E-02 | 2.37E-02 | 5 | grey |
| HALLMARK_MYC_TARGETS_V1 | 50/260 | 200/4383 | 1.06E-19 | 5.21E-18 | 4.82E-18 | 50 | turquoise |
| HALLMARK_OXIDATIVE_PHOSPHORYLATION | 24/260 | 200/4383 | 6.30E-04 | 1.54E-02 | 1.43E-02 | 24 | turquoise |

**
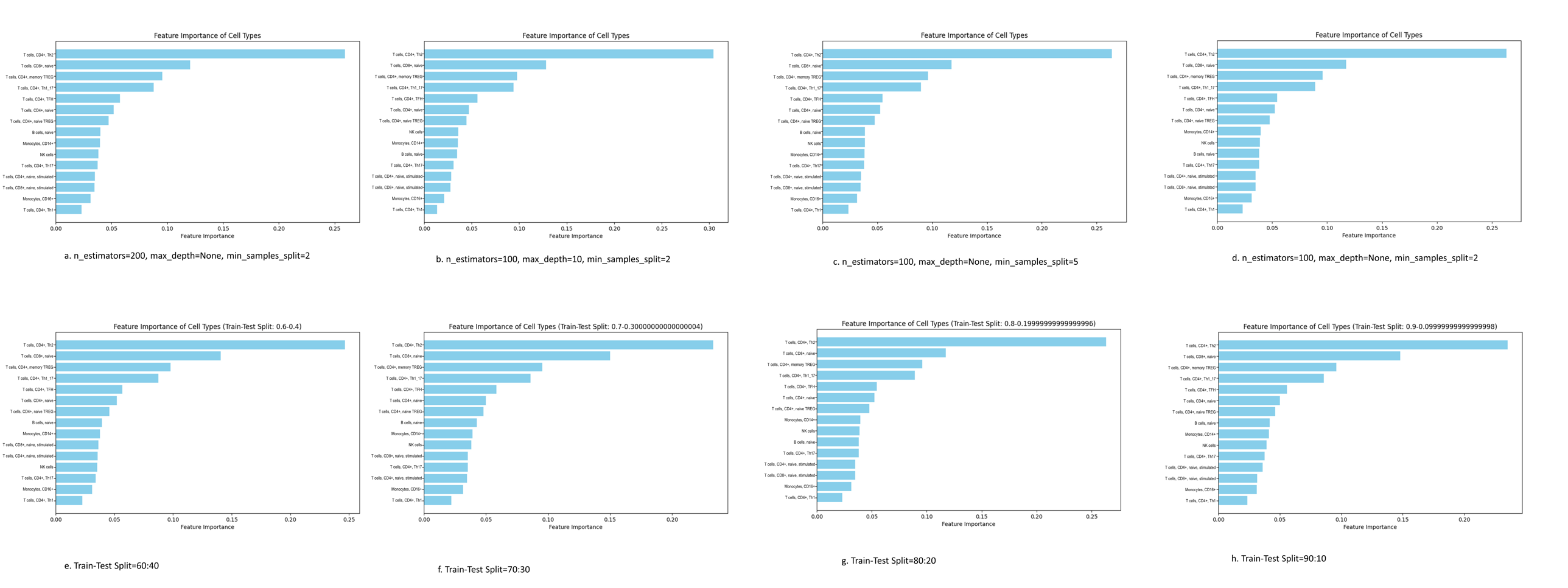
**

**Supplementary Figure 1** Feature importance values of cell types by different RandomForestRegressor parameters. a-d: different n_estimators, max_depth none, min_samples_split. Train-Test Split=80:20; e-h: different train-test split ratio, n_estimators 100, max_depth none, min_samples_split 2. X-axis: Feature importance values (unitless, normalized to sum=1); Y-axis: Cell types.

**
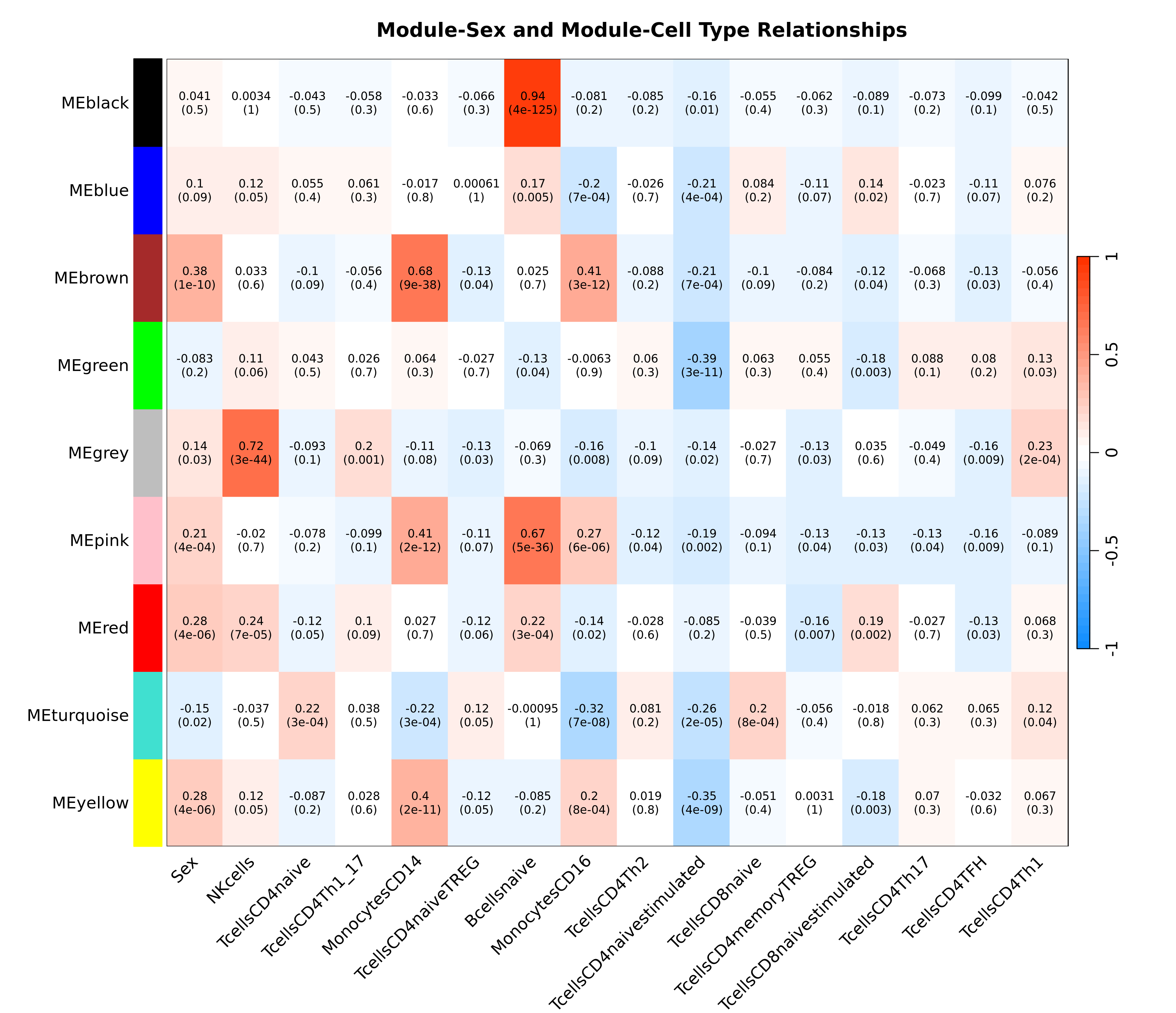
**

**Supplementary Figure 2** Relationships between WGCNA modules and sex/cell type. Numbers indicate correlation coefficients (p-values). Positive correlations with sex indicate higher expression in females, while negative correlations with sex indicate lower expression in females. Across different cell types, the brown, yellow, red, pink, and grey modules show upregulation in females, whereas the turquoise module is downregulated in females. No significant Hallmark (HM) gene sets were identified in the pink and red modules. The turquoise module, enriched for HM_MYC_TARGETS_V1 and HM_OXIDATIVE_PHOSPHORYLATION gene sets, may regulate key pathways for cell growth and energy production. MYC controls genes essential for ribosome biogenesis and cell proliferation, while oxidative phosphorylation supports ATP production, critical for CD8^+^ naïve T cell activation and cytotoxic responses. Its downregulation in females may indicate sex-specific differences in metabolism and growth pathways. In contrast, the grey module, enriched for HM_INTERFERON and ESTROGEN_RESPONSE gene sets, typically expressed at lower levels, shows upregulation in females, likely reflecting enhanced interferon and estrogen signaling in CD4^+^ memory Treg cells, contributing to efficient immune regulation and tolerance. X-axis: Sex and cell types; Y-axis: WGCNA modules; Color legend: Scale from -1 to 1, where values indicate correlation coefficients. Negative values represent downregulation, and positive values represent upregulation.

**
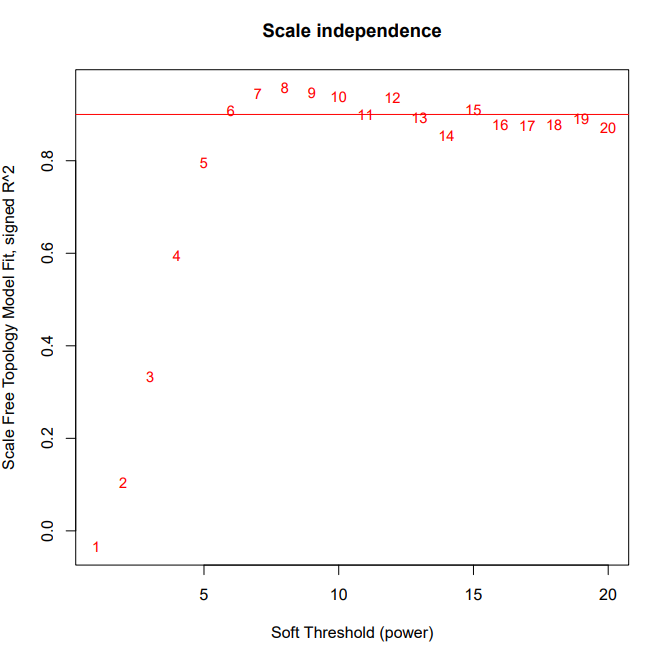
**

**a.**

**
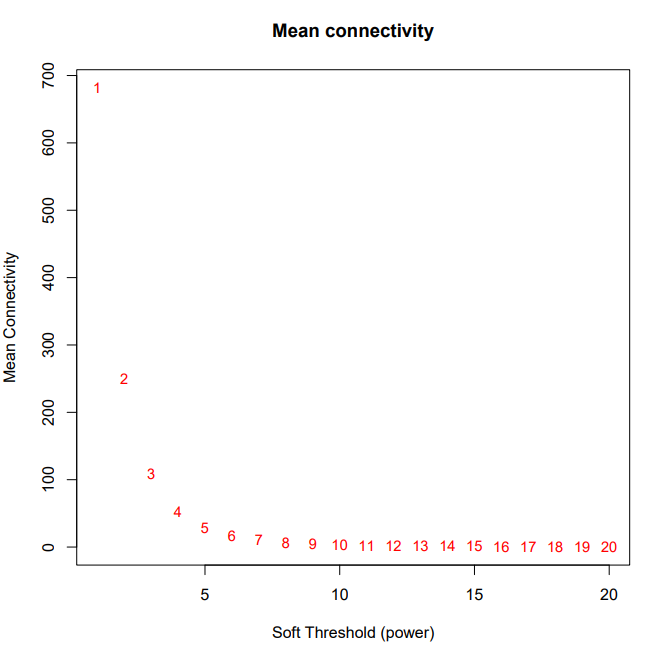
**

**b.**

**Supplementary Figure 3** Connectivity assessment across soft-thresholding powers. (a) Scale-free topology fit index (SFT.R^2) improved with increasing soft-thresholding power, peaking at power 8. X-axis: Soft-thresholding power; Y-axis: SFT.R² (unitless, range: 0–1). (b) Mean connectivity values decreased with increasing soft-thresholding power. X-axis: Soft-thresholding power; Y-axis: Mean connectivity (unitless, approaching zero with increasing soft-thresholding power). Power 7 was selected as it provided an optimal trade-off between network connectivity and scale-free topology.

**Genes within each module and the identified hub genes**

Module: green

Number of genes in module: 175

Connectivity length: 175

Connectivity names: ISG15 AURKAIP1 SSU72 RER1 PARK7 CD52 SYTL1 ATP5IF1 HDAC1 PSMB2 HSPB11 TMEM59 DENND2D PSMB4 KRTCAP2 FDPS PYHIN1 TRAF3IP3 PPP2R5A LINC01871 PTRHD1 XPO1 TMSB10 COX5B DBI MBD5 NMI ATP5MC3 SHISA5 THOC7 ARL6IP5 H1FX SERP1 ATP5ME MXD4 KLF3 RAP1GDS1 HMGB2 NDUFS6 FYB1 PIK3R1 MRPS36 IQGAP2 TBCA RASA1 UQCRQ NDUFA2 ANXA6 LMAN2 TMEM14C DEK HIST1H1C GPSM3 PSMB9 TOMM6 HMGN3 PDSS2 GTF3C6 ABRACL NDUFA4 NT5C3A CCT6A CDK6 LMTK2 POLR2J NDUFB2 GSTK1 AC068587.4 VDAC3 TRAM1 DECR1 PUF60 SEMA4D SEC61B PHPT1 DDIT4 VDAC2 ATP5MD GLRX3 MRPL23 SPTY2D1 UBE2L6 SELENOH TRMT112 CFL1 TBC1D10C CORO1B NDUFS8 CRACR2A MRPL51 ARHGDIB COX14 BIN2 CNPY2 SLC16A7 VPS29 SRSF9 PSMD9 ARL6IP4 DAD1 DHRS4L2 PSME1 PSME2 CHMP4A NEDD8 PSMA6 CALM1 NDUFB1 CRIP1 EID1 RPS27L PSMA4 IDH2 HMOX2 CORO1A BCL7C C16orf87 TMEM208 PSMB6 PFN1 TMEM256 ATP5MC1 PSMC5 ATP5PD NT5C SOCS3 ACTG1 ALYREF MYL12A C18orf32 TXNL1 CNN2 ATP5F1D ABHD17A TIMM13 MAP2K2 NDUFA11 UBL5 DNMT1 ICAM3 NDUFB7 LSM4 REX1BD COPE NDUFA13 HCST PSMD8 TMEM160 SELENOW CARD8 BAX PANK2 TMEM230 DSTN ERGIC3 ROMO1 SAMHD1 SERINC3 PPDPF ATP5PF TIAM1 AP000331.1 SNRPD3 UQCR10 RAC2 SUN2 APOBEC3G C22orf34 CD99 TMSB4X CA5B IL2RG COX7B DOCK11 NDUFA1

Connectivity values: 108.5507 79.6652 88.82394 51.61886 149.7311 332.7521 48.10738 125.3204 58.11904 61.00501 35.87439 140.5855 25.73703 65.16363 99.29782 40.70297 65.05921 90.03456 111.2579 19.07256 36.57734 62.07522 678.435 176.0974 111.8232 113.6818 49.23847 129.8942 46.10959 64.3388 168.8082 133.16 219.3028 147.7728 73.70697 164.3533 90.62702 86.59369 109.692 245.7029 185.8254 56.83601 195.7666 122.5749 96.38835 93.72547 97.35771 54.69031 53.1257 32.35843 157.2392 87.4343 144.4776 93.97536 120.3906 95.78294 62.1555 58.5246 78.88586 207.1027 104.4296 62.14627 71.22619 86.79767 72.31841 117.5585 127.5848 55.24763 56.71396 126.8843 67.68073 53.79876 138.6315 158.5752 41.99214 96.70844 102.9057 123.4557 51.84617 24.82471 85.24877 45.01925 106.5483 80.95112 288.1493 55.88572 73.06333 50.99874 37.87198 42.76385 332.7418 73.66986 117.1745 47.67836 61.91958 76.43892 106.0763 78.61407 151.6094 86.6281 25.12409 193.7274 103.9542 87.26962 106.7389 117.1713 407.8174 81.17332 240.7255 127.9281 78.4438 80.81371 48.72583 47.8631 187.6982 44.62947 50.42745 38.01758 82.28607 353.0823 42.16798 49.19086 69.52176 94.29147 36.60187 36.32438 366.5967 46.84092 266.4784 65.9211 96.11755 86.57046 153.683 86.38099 48.91317 89.16568 108.01 164.0369 57.47682 115.6996 76.25947 59.29463 78.10815 144.2711 190.3852 165.8419 99.72629 60.91997 71.31852 60.01394 68.26748 67.4246 68.81091 82.26405 52.6977 69.83929 161.3412 89.26747 188.0023 118.9295 82.06773 27.85901 49.22691 118.0793 129.5573 88.79738 41.10723 41.5247 155.9694 800.7484 76.96229 125.8743 170.3286 113.7426 149.3992

Hub gene for module green : TMSB4X

Module: turquoise

Number of genes in module: 850

Connectivity length: 850

Connectivity names: MRPL20 RPL22 DDI2 USP48 CDC42 HNRNPR RPL11 SRSF10 SRRM1 SYF2 TMEM50A NUDC STX12 DNAJC8 PHACTR4 EIF3I LCK BSDC1 RBBP4 YARS THRAP3 MRPS15 AKIRIN1 SMAP2 FOXJ3 YBX1 RPS8 UQCRH RNF11 SHISAL2A MAGOH PATJ SERBP1 RABGGTB AC118549.1 USP33 ODF2L GTF2B LRRC8B LRRC8C LRRC8D RPL5 DNTTIP2 CDC14A SLC30A7 S1PR1 PRPF38B PSMA5 CHI3L2 RSBN1 PTPN22 BCAS2 LINC00623 RBM8A LIX1L TXNIP MRPS21 RPRD2 ILF2 JTB C1orf43 ASH1L SSR2 CCT3 IFI16 DCAF8 CD48 PFDN2 UFC1 TMCO1 MPC2 SELL GAS5 CACYBP KIAA0040 SNRPE NUCKS1 FCMR EPRS MIA3 FBXO28 LBR SRP9 PARP1 TOMM20 ADSS CNST RPS7 DDX1 DTNB MRPL33 PPP1CB WDR43 LCLAT1 CEBPZ ZFP36L2 ACYP2 SPTBN1 RPS27A C1D GFPT1 AAK1 GMCL1 SNRPG MPHOSPH10 CCT7 DGUOK IMMT CHMP3 ANKRD36 MGAT4A EIF5B RPL31 NCK2 ZC3H8 BIN1 WDR33 MZT2B R3HDM1 DARS CXCR4 RIF1 CYTIP RBMS1 SSB GORASP2 AC010894.3 HNRNPA3 ORMDL1 AC114760.2 HSPE1 PLCL1 BZW1 CASP8 SUMO1 FAM117B KLF7 CREB1 XRCC5 USP37 EPHA4 DOCK10 MFF SP110 SP100 NCL PTMA ARL4C MTERF4 ING5 CRBN THUMPD3-AS1 SETD5 RPL32 LSM3 CCDC174 NR2C2 CAPN7 OXNAD1 SATB1-AS1 TGFBR2 TRANK1 MLH1 RPSA RPL14 NKTR SMARCC1 MAP4 IMPDH2 IP6K1 GNL3 SPCS1 SFMBT1 CCDC66 FLNB FHIT PSMD6 CGGBP1 CLDND1 CMSS1 FILIP1L SENP7 TRMT10C CBLB TRAT1 CD96 NDUFB4 ZNF148 MRPL3 NCK1 COPB2 SLC25A36 RASA2 RNF7 TFDP2 EIF2A DHX36 SMC4 PHC3 TNIK PIK3CA TTC14 KLHL6 PARL VPS8 DNAJB11 PPP1R2 PAK2 RPL35A TACC3 TNIP2 MRFAP1 BLOC1S4 MED28 KLHL5 AC079921.1 UBE2K PDS5A N4BP2 SLC30A9 TXK OCIAD1 SRP72 REST GRSF1 CCNI CNOT6L PLAC8 HELQ AFF1 PPM1K GPRIN3 DNAJB14 H2AFZ PPA2 AIMP1 LEF1 LEF1-AS1 RPL34 OSTC FAM241A SNHG8 USP53 AC097376.3 INPP4B RPS3A SH3D19 TMEM131L NAF1 CFAP97 SNX25 PDCD6 MED10 ANKH RETREG1 SUB1 IL7R RICTOR PTGER4 RPL37 EMB DHX29 IL6ST SREK1IP1 ADAMTS6 CENPK MCCC2 BTF3 NSA2 RPS23 COX7C TTC37 CAST CAMK4 SRP19 YTHDC2 ATG12 TNFAIP8 CEP120 GRAMD2B SLC12A2 RAPGEF6 HSPA4 VDAC1 UBE2B SAR1B CAMLG DDX46 HNRNPA0 HSPA9 PAIP2 UBE2D2 IK TAF7 NDFIP1 LARS ITK AC010609.1 TTC1 NPM1 SFXN1 PRR7 HNRNPAB EXOC2 RIOK1 TMEM14B HIVEP1 NOL7 HIST1H4C HLA-A ZNRD1 HLA-E ABCF1 HLA-C LTB PSMB8 CUTA HMGA1 RPS10 RPL10A STK38 MTCH1 CCND3 RPL7L1 HSP90AB1 SUPT3H SMAP1 EEF1A1 COX7A2 PNRC1 BACH2 CRYBG1 FYN TSPYL1 KPNA5 THEMIS TNFAIP3 AL031772.1 HIVEP2 SF3B5 PPIL4 MTHFD1L OPRM1 IPCEF1 DYNLT1 SYTL3 PRKN FAM120B PSMB1 PDCD2 COX19 FOXK1 RPA3 GLCCI1 ARL4A SP4 CYCS RALA PSMA2 STK17A COA1 MRPS24 PPIA SNHG15 IKZF1 SEC61G CHCHD2 TYW1 BUD23 MDH2 RSBN1L DMTF1 TMEM243 ANKIB1 AC079781.5 PRKRIP1 LSM8 ZNF800 UBE2H CHCHD3 ZC3HAV1 SSBP1 TRBC1 TRBC2 GIMAP7 GIMAP4 GIMAP5 UBE3C MTMR9 CHMP7 CCDC25 SARAF AC044849.1 LEPROTL1 AC026979.2 DCTN6 NSD3 GOLGA7 KAT6A FNTA RB1CC1 CHD7 SNHG6 ELOC RBIS WWP1 CPNE3 UQCRB COX6C EIF3E EIF3H AC104316.1 AC016074.2 LINC00861 MYC PUM3 CDC37L1 RCL1 MLLT3 TOPORS SMU1 BAG1 TOMM5 CEMIP2 AUH IARS ERCC6L2 ANP32B UGCG TNFSF8 RC3H2 RABGAP1 RPL35 SCAI RPL12 C9orf16 FNBP1 FUBP3 AL157938.2 GTF3C4 RPL7A SURF4 EDF1 DPP7 MRPL41 GTPBP4 PRKCQ PRKCQ-AS1 ATP5F1C CDC123 FAM107B TRDMT1 ZEB1 EPC1 CUL2 CREM ZNF33A ZNF37A HNRNPF ZNF22 MAPK8 ANK3 ARID5B SLC25A16 PPA1 SPOCK2 ANAPC16 ANXA7 RPS24 CCSER2 KIF20B NDUFB8 TRIM8 SMC3 ABLIM1 GRK5 PPP2R2D MTG1 PSMD13 IFITM1 CD81 CARS TRIM5 EIF3F RPL27A TMEM41B COPB1 PSMA1 PDE3B C11orf58 LDHA EIF3M CAPRIN1 FNBP4 UBXN1 SF1 VPS51 PACS1 RBM4 MRPL21 AP002336.2 RNF169 SPCS2 CLNS1A CWC15 FAM76B MAML2 BIRC3 TMEM123 FDX1 RBM7 PCSK7 MPZL3 CD3E CD3D ATP5MG KMT2A DDX6 SC5D SRPRA ETS1 ZBTB44 WNK1 CCND2 C12orf57 NECAP1 CLEC2D CLEC2B YBX3 DUSP16 CDKN1B STRAP GOLT1B LDHB CCDC91 SINHCAF RESF1 NELL2 PCED1B PCED1B-AS1 ATP5MC2 HNRNPA1 TESPA1 PA2G4 RPL41 PTGES3 NACA MARS DDIT3 DYRK2 YEATS4 CCT2 TBC1D15 ATXN7L3B KRR1 CCDC59 LINC01619 BTG1 AC025164.1 VEZT METAP2 APPL2 BTBD11 PWP1 RPL6 BICDL1 COX6A1 TRIAP1 DYNLL1 TMEM120B RHOF TMED2 UBC RAN MPHOSPH8 SAP18 GTF3A POMP NAA16 TPT1 COG3 ZC3H13 ESD PHF11 RNASEH2B COMMD6 TPP2 ARHGEF7 RASA3 APEX1 PNP SUPT16H TRAC NGDN IRF9 EAPP SNX6 BAZ1A SRP54 FAM177A1 PPP2R3C PNN PRPF39 RPS29 RPL36AL RTRAF TXNDC16 CNIH1 PSMA3 PRKCH HIF1A-AS3 SYNE2 CHURC1 MAX DCAF5 ERH COX16 TMED10 SNW1 CCDC88C TC2N PAPOLA BCL11B EVL PPP2R5C HSP90AA1 ATP5MPL SNRPN APBA2 NSMCE3 RASGRP1 SRP14 RTF1 AC068724.3 CTDSPL2 COPS2 GABPB1-IT1 RSL24D1 RNF111 RORA USP3 USP3-AS1 HERC1 PPIB PDCD7 DPP8 HACD3 ISG20 NGRN CRTC3 LINC01578 SNHG9 RNPS1 IL32 NLRC3 ATF7IP2 ZC3H7A RSL1D1 AC007216.4 PDXDC1 MARF1 ABCC1 RPS15A THUMPD1 POLR3E AC092338.3 NDUFAB1 XPO6 LAT YPEL3 DNAJA2 ITFG1 LONP2 CYLD RBL2 NLRC5 CIAO2B CFDP1 APRT SPG7 RPL13 ITGAE RABEP1 C1QBP DERL2 NLRP1 C17orf49 EIF5A ACAP1 RPL26 TTC19 MAP2K3 FAM222B ABHD15-AS1 SSH2 CRLF3 LRRC37B PSMD11 TAF15 DDX52 RPL23 RPL19 IKZF3 CCR7 KRT10 DNAJC7 LSM12 KPNB1 SPOP TOB1 NME2 SRSF1 LIMD2 CCDC47 SNHG25 PRKCA RPL38 SUMO2 UBALD2 SNHG16 MFSD11 TMC8 NDUFAF8 CD7 AP001011.1 PSMG2 LDLRAD4 SNRPD1 TTC39C TAF4B RNF125 MAPRE2 ATP5F1A C18orf25 MEX3C MALT1 LMAN1 BCL2 VPS4B TXNL4A DAZAP1 RPS15 TLE5 MRPL54 EEF2 RPL36 ALKBH7 PET100 MAP2K7 RPS28 EIF3G ZNF433-AS1 TRIR CALR C19orf53 IL27RA RASAL3 KLF2 RPL18A ARRDC2 UBA52 PBX4 FXYD5 EIF3K RPS16 PSMC4 RABAC1 TRAPPC6A SNRPD2 NOP53 RPL18 SNRNP70 NOSIP PRMT1 ZNF480 ZNF83 RPS9 RPL28 A1BG CSNK2A1 SNRPB NOP56 SHLD1 TASP1 SNRPB2 SMIM26 CRNKL1 XRN2 NXT1 MAPRE1 CBFA2T2 EIF2S2 TRPC4AP SCAND1 STK4 SLC35C2 ELMO2 RAB22A STX16 GNAS RPS21 PRPF6 BTG3 CCT8 BRWD1 RRP1B UBE2G2 SUMO3 PRMT2 UFD1 DDT MTFP1 PIK3IP1 PIK3IP1-AS1 SFI1 HMGXB4 APOL3 MFNG TOMM22 RPS19BP1 SNU13 SMDT1 NUP50 ATXN10 AL118516.1 PIM3 AKAP17A OFD1 SYAP1 SCML1 EIF1AX EIF2S3 PDK3 NDUFB11 RBM3 PQBP1 PIM2 NBDY FAAH2 IGBP1 RPS4X P2RY10 GPR174 ITM2A RPL36A TSC22D3 SLC25A5 UPF3B RPL10 MT-ND6

Connectivity values: 71.97838 598.4145 71.20326 76.96755 391.2503 166.7418 829.6065 229.4729 285.6872 198.1049 162.3886 79.25967 91.43054 136.2385 90.83894 95.16638 95.34596 105.1789 113.6537 158.2563 215.0987 60.56346 118.3013 364.6689 160.1467 404.1324 841.6336 218.1368 91.25448 38.6519 109.6589 59.534 226.2091 48.02714 92.19655 111.7276 89.23073 143.0627 66.70194 138.1678 93.60715 677.5447 87.85237 301.4451 87.53908 55.07738 149.0876 80.66068 15.59129 92.5001 91.84307 156.4416 50.38297 166.4601 30.633 485.6739 76.02128 115.194 82.35707 126.9963 83.61071 290.2371 173.9323 76.10478 135.387 80.12846 236.2477 104.69 128.9507 74.47133 89.86431 99.54503 291.8888 75.14373 26.40762 98.9686 162.5765 66.72521 53.81201 91.03106 71.50643 105.01 91.76372 52.36136 218.2706 70.37473 146.4457 713.5088 49.2902 58.31788 75.45356 252.3087 119.1001 56.39099 118.5006 318.9464 77.38154 133.2452 831.8821 90.0946 63.54707 220.8802 60.12065 148.194 55.63803 56.67066 81.33282 75.18467 104.796 60.36594 149.0241 127.5924 491.6509 141.491 53.11093 71.73535 162.2571 185.6217 72.78478 73.74804 293.1166 138.8786 283.2507 214.241 97.82765 68.16595 71.15156 256.1008 110.0252 56.83721 119.4376 136.3393 207.8291 168.1633 148.4543 138.8332 86.91941 118.4479 189.8268 63.19168 80.85944 285.3657 78.50991 129.3552 255.5215 263.3341 681.5245 244.9587 91.43489 44.48953 127.7526 115.8004 131.8709 847.4909 79.63159 65.27968 100.7565 98.68981 165.0368 43.9284 248.0103 71.13393 35.31784 611.5956 669.4766 182.9269 190.4347 92.90309 39.0055 141.9622 76.60426 118.609 93.5732 78.92568 70.03277 124.8437 56.22775 100.8363 99.99907 163.9731 101.1279 109.0375 79.50152 259.8593 102.8761 198.5697 123.224 180.34 52.67136 95.08926 47.56337 120.0707 268.0186 121.7894 137.0471 122.6298 162.451 101.7413 165.7363 183.9792 225.728 81.36588 84.21934 61.92011 80.41698 64.05361 217.2147 213.1899 709.3396 45.65813 47.44716 92.43615 40.44331 64.69489 153.9941 49.16535 198.3377 184.9905 68.48133 60.2105 133.6948 112.3507 112.851 84.80938 57.6651 328.4805 370.311 159.7262 32.19835 144.4625 61.65035 203.4723 118.5537 155.9806 52.43028 122.3932 211.0061 41.8416 825.848 70.64995 58.40911 244.855 43.48115 48.68143 130.9922 803.6834 73.59455 235.0008 49.59614 43.50791 89.07414 67.95534 122.1437 86.23394 75.14038 335.448 331.2094 282.5928 74.81485 782.3706 129.8524 63.54266 211.5847 96.96759 24.50529 48.04484 38.34763 426.678 211.465 802.7856 346.4958 78.88212 284.8611 297.9767 114.8042 93.63846 85.48093 246.4798 54.82827 56.2728 49.93278 272.6327 67.5761 84.61112 261.6628 69.71998 112.818 130.3702 135.2947 156.7371 233.9025 273.6529 121.58 97.65635 284.794 45.45111 225.3111 91.34403 76.91255 402.4569 94.02665 36.69347 69.55161 80.54499 69.78149 93.86031 198.1567 140.663 108.1865 538.7153 43.66031 417.577 139.2747 526.7786 354.6877 117.0323 112.9814 66.2893 397.3555 650.6684 162.2944 118.7018 288.5588 69.25057 384.4972 86.59325 130.7369 941.6322 187.8202 401.3655 390.4302 212.3212 406.4749 52.80732 64.47199 85.8368 421.2137 57.6114 201.7613 143.2571 95.30628 59.65914 31.5074 74.94992 148.6316 186.9373 30.11841 71.93354 152.1006 56.14906 96.17966 97.14987 53.68469 95.49238 76.87616 58.44078 169.1556 199.566 130.3615 351.4067 135.6186 79.97793 311.2555 88.33645 281.4387 139.778 312.4604 82.46738 86.48942 84.60012 90.4612 75.1762 152.4325 90.28637 61.21823 102.1184 101.7638 96.52566 248.7271 79.84678 262.0526 124.5106 122.0139 190.5796 139.7255 58.81708 43.55752 85.82803 46.62104 62.2178 37.24365 479.4713 90.11308 303.218 118.228 65.30952 196.0806 58.40778 310.9134 91.13934 260.4952 126.4335 253.5446 109.0318 97.38666 82.558 43.07752 341.0177 221.5141 297.0765 276.4681 41.43177 29.06738 77.83007 47.06049 61.59267 42.90101 48.63702 142.9256 55.27195 95.23563 80.47907 90.11271 243.7712 52.07717 82.86143 73.12168 228.5585 73.84869 78.08524 92.36818 111.6918 605.9456 37.8501 721.1347 143.746 359.1291 48.86581 77.08069 27.10856 703.1323 98.6557 180.1243 70.10878 63.09209 70.90035 143.9829 121.8451 113.6835 93.42803 323.4559 31.35594 408.656 328.9167 89.36857 167.2215 102.8576 55.24957 132.6257 40.30383 211.0011 147.5529 206.0026 64.34246 116.9017 166.9404 148.0286 111.248 781.3811 247.5029 90.52308 134.1077 68.17555 115.3033 198.9213 194.3718 110.762 42.08831 68.5155 82.23475 92.62487 54.87973 25.13066 224.1966 659.13 106.6986 101.1667 175.1678 387.768 201.9089 166.6167 167.2874 84.76991 204.0355 169.6017 303.4944 92.51927 225.1857 144.3141 53.4397 53.12444 79.45541 129.8509 66.55133 107.4525 64.968 233.911 161.4356 252.3585 57.34523 55.78753 94.87933 68.30761 168.5419 206.4647 326.1958 142.9537 248.463 44.32184 58.65183 293.8135 80.6251 189.4657 90.71278 210.6632 66.73236 204.8439 186.933 143.4125 112.1917 67.58528 143.8064 60.28947 223.3878 204.8178 96.56901 130.5136 133.1434 124.685 205.7694 257.9633 355.3331 98.01015 117.8082 894.4979 196.2483 622.68 68.43877 81.39246 51.90351 44.51477 103.349 207.8943 77.00465 123.8576 160.7032 392.6908 572.8374 53.38697 111.7252 117.7012 73.91882 105.8687 59.0915 715.5841 171.1884 167.5995 38.02007 78.36603 34.67799 54.95637 69.01009 487.3124 152.5484 118.7907 209.864 170.2502 173.9229 73.6189 940.0437 75.8104 86.71866 48.62043 76.12453 64.94583 246.0992 155.6801 166.3211 253.2225 62.62582 39.67607 82.11138 113.1831 57.86487 77.11633 157.1807 159.9558 232.795 91.50134 172.9824 96.69305 253.1642 59.57106 778.5624 355.8242 131.4226 60.19928 53.64928 100.7368 331.1455 59.7905 259.3897 134.1194 184.5929 83.60562 106.4629 82.27434 94.0954 136.6635 160.9974 129.3654 167.0353 247.1363 219.9024 359.6184 451.0112 171.403 59.75115 103.0357 79.12495 148.474 317.2998 144.7629 50.31778 142.4991 117.5721 85.90483 192.4332 127.1784 244.6207 344.7393 170.0406 387.7751 177.1236 44.17803 74.99888 46.07794 189.4585 39.65745 134.1126 375.0816 107.893 112.4378 235.6151 52.06532 108.2748 152.9961 142.8852 63.25344 113.9271 94.98594 229.2724 821.6633 84.5097 75.64422 51.22557 67.41671 143.0611 56.54052 108.7643 84.85283 152.6469 105.3316 226.1572 123.8306 137.4403 79.34747 96.3496 173.2586 94.5036 966.1099 66.32284 137.2598 95.32811 57.90195 175.8816 99.26244 173.8789 107.2955 780.1514 109.9015 88.49979 119.7713 56.94922 373.0979 96.34193 49.32731 73.47652 117.952 70.57907 540.9535 777.379 139.8142 137.9993 125.7107 135.6674 66.92482 128.7236 64.55187 85.29766 225.3657 56.91845 213.4857 86.9141 89.8822 194.8511 569.7029 232.7226 169.672 91.43494 62.9884 88.08093 54.58771 126.1111 211.7119 72.81179 176.1002 115.9906 148.2645 100.3366 206.5413 149.319 104.4677 106.8248 55.62356 218.3845 65.29517 222.9393 127.5911 99.04103 71.33739 727.9124 269.6339 111.3343 340.183 645.8858 79.44393 125.1997 42.62925 767.1786 170.3746 81.13171 196.0478 157.2894 145.4625 61.80141 62.23951 279.0074 797.3532 59.71362 651.069 142.8025 215.4277 268.4334 695.5232 58.22063 102.9254 49.43577 183.3704 294.609 715.4562 104.9539 149.7686 47.37805 34.02969 55.89634 692.4759 852.4367 25.75788 82.03024 177.4373 43.74038 53.27006 57.59379 117.0138 87.31944 44.04922 167.5749 67.94103 101.6941 89.37658 133.6056 121.4487 111.4068 330.3414 46.21233 47.57678 127.2133 80.13392 419.5648 677.5118 71.03279 107.7781 92.0594 167.9146 69.95538 79.39747 81.92368 149.013 79.90496 98.34024 33.02134 211.9042 78.13505 80.49951 80.89103 62.35075 54.55986 72.22514 89.7891 196.3434 126.1052 70.72474 83.55199 52.4029 109.8606 57.9806 81.81009 156.0108 77.56727 147.2478 151.3878 70.13479 146.4153 234.8658 52.95363 66.40372 103.4534 117.1385 104.3137 751.7403 65.50488 56.1175 114.4481 590.2026 337.4023 174.9248 58.68417 924.2003 103.9542

Hub gene for module turquoise : RPL13

Module: grey

Number of genes in module: 71

Connectivity length: 71

Connectivity names: ATAD3B EYA3 ZMYM4 KIAA0319L IFI44 CD160 C1orf21 LAX1 RGPD2 IFIH1 IKZF2 AGAP1 SNED1 CMC1 ATP2C1 SIAH2 TFRC AREG TSPAN5 HPGD IRF2 CASP3 GZMK CYSTM1 N4BP3 SOX4 NCR3 UBR2 SLC35F1 MYB AHI1 TYW1B PPP1R9A LRRN3 FDFT1 TOX MTFR1 ZNF704 SPAG1 MAF1 DDX58 STAM PIP4K2A RTKN2 IFIT2 IFIT3 ITPRIP PNPLA2 DDB1 SYTL2 SESN3 LINC02446 FGFR1OP2 CRADD RGCC IGF1R RANBP10 SLC7A5 SREBF1 CCL5 NBR1 DGKE TANC2 NFATC1 ADAMTS10 PSMF1 SRSF6 ZMYND8 CYSLTR1 PRKY USP9Y

Connectivity values: 20.70906 47.44834 54.92547 50.20533 38.17743 6.305495 28.48829 29.24879 19.09054 68.00588 75.32676 14.30411 50.19355 41.21425 82.877 74.76441 77.23126 48.93932 65.7087 72.11206 41.52426 52.48543 39.53757 74.82457 12.19087 64.05309 26.02455 114.9177 44.45784 12.06185 53.20992 41.31046 8.720157 19.68328 63.41744 117.5453 45.54572 40.48715 40.13267 65.64147 45.7237 105.3711 210.6882 40.91648 36.69527 32.11441 57.16198 47.27972 71.02544 41.85978 127.2974 48.23815 93.69427 39.10408 125.2624 75.67768 66.34682 82.44455 41.84277 127.2601 44.27883 33.7038 47.96081 63.10397 12.56434 100.304 73.35062 58.91051 29.14496 54.77708 62.5225

Hub gene for module grey : PIP4K2A

Module: blue

Number of genes in module: 611

Connectivity length: 611

Connectivity names: CDK11B CDK11A GNB1 CAMTA1 SLC25A33 UBR4 EIF4G3 LUZP1 RUNX3 MACO1 PUM1 SFPQ SNIP1 MACF1 RLF NFYC FAF1 EPS15 TUT4 USP24 MYSM1 JAK1 PDE4B SLC35D1 SRSF11 ZRANB2 SELENOF PKN2 ZNF644 MTF2 MFSD14A VAV3 HIPK1 MAN1A2 ENSA GATAD2B COPA UHMK1 PRRC2C SUCO KLHL20 RABGAP1L COP1 TOR1AIP1 DHX9 ODR4 DENND1B PTPRC KDM5B ADIPOR1 RAB3GAP2 DUSP10 TP53BP2 ACBD3 ARID4B AKT3 AHCTF1 TRIM58 NOL10 NBAS ATAD2B ITSN2 ATL2 SOS1 EML4 PPM1B FBXO11 PSME4 PPP4R3B REL USP34 WDPCP MDH1 UGP2 PELI1 PPP3R1 ZNF638 EXOC6B KCMF1 STARD7 TMEM131 CCDC93 CLASP1 AC068282.1 SMPD4 MGAT5 CCNT2 RAB3GAP1 ARHGAP15 AC079793.1 PRPF40A TANK PSMD14 PPIG HAT1 SP3 OLA1 WIPF1 ITGA4 STK17B ANKRD44 MOB4 CLK1 ORC2 TRAK2 CUL3 DIS3L2 ILKAP HDAC4 SUMF1 RAF1 ANKRD28 RFTN1 PLCL2 RAB5A UBE2E1 STT3B CMTM7 CLASP2 GOLGA4 WDR48 SNRK CCDC12 SETD2 KLHL18 IP6K2 QRICH1 RBM6 DCAF1 DCP1A ATXN7 SLC25A26 SUCLG2 UBA3 FOXP1 ZNF654 MTRNR2L12 ZBTB11 CD47 USF3 NAA50 ZBTB20 GSK3B SNX4 ZXDC RAB7A RYK STAG1 ARMC8 ANKUB1 CCNL1 PRKCI TBL1XR1 FXR1 ATP11B KLHL24 SENP2 TRA2B EIF4A2 SENP5 DLG1 LRCH3 GAK FAM193A HTT DHX15 STIM2 RELL1 TBC1D1 RFC1 RHOH FRYL CENPC UBA6 YTHDC1 SNCA EIF4E SLC39A8 UBE2D3 SEC24B MCUB SMARCA5 OTUD4 ARHGAP10 LRBA AC023424.3 ETFDH SDHA MARCH6 OTULINL ZFR ZNF131 PARP8 MTREX PDE4D BDP1 CCNH FAM172A CHD1 PAM PPIP5K2 MAN2A1 REEP5 CSNK1G3 CDC42SE2 FNIP1 AFF4 PPP2CA KDM3B ANKHD1 ZMAT2 RBM22 DOCK2 FBXW11 NSD1 HNRNPH1 GMDS RIPK1 PRPF4B CDYL RANBP9 MYLIP GMPR RIPOR2 HIST1H1E HLA-F TRIM26 TAPBP ILRUN ZFAND3 BTBD9 TMEM63B CD2AP FBXO9 LMBRD1 SENP6 PHIP SYNCRIP ATG5 SESN1 NUS1 CEP85L SERINC1 MED23 REPS1 UTRN STXBP5 GINM1 LATS1 SCAF8 ARID1B SNX9 SYNJ2 EZR EIF3B CARD11 USP42 BZW2 SNX13 TRA2A KBTBD2 ELMO1 CDK13 LANCL2 ZNF92 AC068533.3 EIF4H CYP51A1 SEM1 TRRAP PILRB POLR2J3 SRPK2 PNPLA8 IMMP2L ING3 NRF1 AC016831.7 LINC00513 BPGM LUC7L2 BRAF EZH2 GALNT11 RBM33 DNAJB6 ESYT2 MCPH1 TNKS BNIP3L GTF2E2 POLB ATP6V1H PDE7A ARFGEF1 ZBTB10 PAG1 RNF19A YWHAZ UBR5 TAF2 ZHX2 ATAD2 PVT1 EFR3A AGO2 RFX3 KIAA2026 KDM4C DENND4C UBAP1 PTAR1 SMC5 SECISBP2 MFSD14C PSMD5 RAB14 STOM PSMB7 ARPC5L SPTAN1 NUP188 C9orf78 PRRC2B MED27 EHMT1 KLF6 RBM17 CELF2 UPF2 CAMK1D ABI1 YME1L1 RAB18 CCNY NCOA4 JMJD1C HNRNPH3 ECD NUTM2B-AS1 BTAF1 MARCH5 EXOC6 HPS1 CHUK FBXW4 OGA NFKB2 MXI1 DUSP5 VTI1A FAM160B1 AP2A2 HBB EIF4G2 RRAS2 PIK3C2A CD44 TTC17 ATG13 RASGRP2 ATG2A NDUFV1 RSF1 CHORDC1 TAF1D CUL5 NPAT PTS SIK3 ARCN1 CHD4 BORCS5 ETNK1 YAF2 SLC38A1 CCNT1 PCBP2 R3HDM2 MON2 LEMD3 HELB RAP1B FRS2 CNOT2 ZFC3H1 ZDHHC17 CDK17 GNPTAB CRY1 PPTC7 SPPL3 KDM2B PSPC1 ZMYM2 XPO4 ZDHHC20 MICU2 NUP58 PAN3 USPL1 N4BP2L2 FOXO1 DLEU2 KLF12 MYCBP2 RBM26 UBAC2 PCCA ARGLU1 HNRNPC ACIN1 ARHGAP5 RALGAPA1 FBXO33 NEMF SOS2 MAP4K5 GNG2 DDHD1 MAPK1IP1L PSMA3-AS1 ARID4A PPP2R5E ZBTB1 RBM25 ZNF410 AC005480.1 FOXN3 PPP4R3A TRIP11 DDX24 CCNK DYNC1H1 WDR20 TRAF3 KLC1 GPR132 NIPA2 UBE3A KLF13 EMC7 AQR INO80 TMEM87A B2M SPPL2A TMOD3 RAB8B ZNF609 RAB11A PIAS1 MYO9A ARIH1 SIN3A PEAK1 IREB2 ZFAND6 WHAMM BTBD1 HBA1 HBQ1 LUC7L SRRM2 CORO7 GLYR1 USP7 C16orf72 CLEC16A NDE1 SMG1 CDR2 RBBP6 TNRC6A IL21R FUS TENT4B IST1 USP10 BANP ZFPM1 ANKRD11 AC092120.3 TCF25 YWHAE SMG6 METTL16 UBE2G1 PER1 UBB MPRIP RFFL GGNBP2 CWC25 STAT5B SLC25A39 EFTUD2 CALCOCO2 LUC7L3 TSPOAP1-AS1 RPS6KB1 BCAS3 TLK2 DDX5 CEP95 HELZ PITPNC1 BPTF SAP30BP SRSF2 CYTH1 USP36 RNF213 CCDC57 FOXK2 YES1 SMCHD1 PTPN2 ESCO1 SS18 ZNF24 PIK3C3 SMAD2 DYM RELCH ZNF407 TSHZ1 MBP PTBP1 ARHGAP45 YJU2 LONP1 ILF3 ZNF44 RAD23A ADGRE5 MED26 FKBP8 ZNF506 BLVRB TGFB1 AC011476.3 ZNF586 PTPRA ATRN SMOX SLC23A2 CDS2 BCL2L1 RALY RBM39 TGIF2 RALGAPB PPP1R16B STAU1 ADNP DIDO1 USP25 PAXBP1 SON TTC3 DYRK1A ZBTB21 ABCG1 NDUFV3 PCNT MED15 PI4KA PITPNB EWSR1 PISD FBXO7 DDX17 JOSD1 TNRC6B RBX1 SREBF2 BRD1 PPP6R2 PRKX PRKX-AS1 TBL1X TXLNG SH3KBP1 RPS6KA3 ZFX MED14 USP9X DDX3X KDM6A OTUD5 SMC1A FTX JPX RLIM ATRX BRWD3 MORF4L2 RBMX MT-ND1 MT-ND2 MT-CO1 MT-CO2 MT-ATP8 MT-ATP6 MT-CO3 MT-ND3 MT-ND4L MT-ND4 MT-ND5 MT-CYB

Connectivity values: 83.73339 103.5248 286.4448 155.0542 69.89804 127.5116 106.417 92.90339 189.3933 155.697 243.7316 274.3202 78.6969 191.6403 213.5928 59.22018 194.9601 178.7167 260.4961 153.4564 71.0351 404.1254 167.8928 71.08177 280.0254 167.1887 131.9506 172.2659 310.1331 117.2901 120.243 158.0847 170.1594 112.904 177.6024 217.8182 149.0582 80.58137 433.455 138.504 72.37879 299.6781 250.0962 94.61853 190.4094 67.54855 105.1206 486.9254 83.57291 87.06364 94.86874 66.72549 137.7806 106.8511 496.0543 190.467 127.8037 46.47092 99.56254 94.12999 163.9799 248.4698 85.04232 172.981 421.1938 195.5509 225.0106 200.8995 162.9395 285.4871 286.9636 76.015 62.27953 63.38609 252.1462 164.9487 142.4525 84.30163 157.7766 72.25335 124.845 107.5848 193.054 124.4291 53.9687 172.6465 89.58997 165.7445 489.6884 191.1916 194.861 275.1624 87.16086 196.6413 52.00104 200.6496 118.2807 230.3495 179.529 373.2911 310.8192 97.57977 185.4102 59.39166 64.94901 335.8938 74.82104 107.5198 98.51505 67.55252 133.3426 126.7222 163.6618 168.7666 131.3865 165.0132 178.0585 68.1575 150.2691 255.0822 157.2349 163.722 154.6192 268.8269 66.34187 93.97995 117.6475 167.9998 81.64844 165.5782 128.4268 140.5278 112.8984 52.86379 474.0506 74.47182 444.3688 103.1232 132.7504 83.00896 97.20827 256.5551 162.8785 78.75098 70.81719 254.8667 96.99811 332.0869 89.73216 46.85627 329.0624 47.05987 216.6365 176.7077 256.0646 195.8849 101.177 258.068 197.0928 184.2506 116.2168 105.4307 94.46552 88.77033 110.7866 90.85407 136.6424 86.51062 93.39494 141.9977 346.9821 128.1747 219.2815 109.087 226.7232 88.25854 103.2922 45.92713 360.6069 139.1984 180.4929 136.4061 65.08491 52.96517 247.5195 75.18656 58.35889 71.98556 231.7264 54.26271 167.6077 120.4447 247.6603 121.9694 244.2079 115.4238 197.4824 240.017 318.9948 70.44424 69.75232 151.3551 76.76324 123.6581 519.3208 209.692 209.4483 165.7761 100.5873 228.6761 71.76422 68.23131 232.1649 128.5047 105.897 191.8804 106.8524 93.16325 208.8489 99.12612 139.606 138.9219 34.43778 278.3417 61.69855 130.4838 67.11988 174.5842 85.13444 342.2901 265.3829 53.8889 136.4775 55.2393 173.9397 159.5504 159.6075 135.3562 104.6942 85.38072 83.05631 159.7454 171.9323 84.21302 96.75718 306.9353 73.51305 87.91105 96.42894 184.0514 281.2402 367.8147 65.11471 318.4637 77.75225 157.1021 84.64814 58.21147 82.01506 267.6023 107.6154 230.3182 179.0057 71.97162 54.07766 28.97624 128.7579 48.1066 80.21817 91.88798 58.77353 96.90088 212.0098 128.2604 113.8029 70.6055 125.3898 113.488 178.6985 83.95934 242.2965 326.6504 125.6934 89.73377 209.1448 230.8902 239.1431 83.68538 154.7564 184.6016 57.9003 60.98963 113.4649 153.9251 200.7236 100.8465 196.8946 239.8384 371.0124 229.8648 109.5706 222.1062 80.82222 94.27034 144.3828 175.7395 128.7465 87.1608 153.929 122.3813 199.7911 81.288 161.0686 173.3388 90.08583 86.27322 97.12757 47.22129 127.8839 92.7802 107.3183 33.14258 179.4378 184.7845 83.89041 151.4542 367.4708 123.6825 383.0863 115.1756 313.5957 203.8512 260.0941 110.1626 203.5833 134.8791 381.1214 174.8731 91.56224 118.1016 163.5073 92.08043 75.60433 46.09865 41.4285 68.03528 174.4274 93.17258 118.3649 32.85368 155.6033 66.78214 57.15444 364.9771 209.1257 121.1663 126.301 378.2207 154.6789 107.6436 80.14594 82.71185 57.19506 212.0398 63.73232 174.1404 151.9907 75.19646 73.57673 415.3402 64.0823 129.7691 83.87115 89.80048 183.2361 342.4619 100.98 229.6454 143.1584 173.6115 58.9531 109.2031 408.7011 115.146 248.6231 175.3514 79.44278 241.2754 108.5075 164.7057 81.34238 183.7522 107.5638 167.8607 161.8329 160.345 196.4258 149.5199 110.5932 307.8037 83.25557 248.6828 243.8454 195.9706 264.4947 214.9713 142.8629 194.2693 47.79632 228.0137 411.3112 120.749 57.03097 182.5835 122.6841 149.031 164.6143 95.93929 353.6505 138.5275 151.0543 101.6439 129.6467 133.7326 162.6992 241.5203 87.41482 47.60611 309.8478 174.4187 45.38549 276.483 118.1539 131.1379 107.6779 273.5054 106.4516 102.595 61.92714 165.6878 165.6369 71.71567 104.8839 121.4717 168.4433 925.7932 158.9594 161.4086 285.7259 91.3839 327.3423 267.4464 57.2333 352.6402 99.86182 51.41221 81.33013 238.3008 254.0316 80.61948 282.4713 26.3269 113.7452 309.3052 170.7637 92.15989 127.0901 53.94009 76.52543 109.0184 168.8849 126.0499 139.1306 210.5324 94.05512 279.289 84.35679 122.0709 139.5924 68.81585 52.2313 384.3282 59.83431 217.3891 169.0736 145.0086 107.3506 145.4308 145.2264 383.9291 59.2389 121.5131 178.3582 85.45627 126.6168 78.33864 69.65806 90.58085 222.2165 77.00987 129.3328 135.0691 163.7588 538.6121 95.10827 216.6113 304.0042 220.16 119.7863 171.0692 343.0834 168.8404 201.4842 75.22008 103.6689 125.0643 455.9945 169.7703 103.742 96.58247 67.65532 69.04101 90.40263 133.7909 68.14681 125.8746 38.83566 216.4137 73.52574 93.54297 48.31826 39.08351 146.6368 63.46021 95.50625 276.3886 49.25625 130.274 83.63865 91.85504 229.202 75.92482 58.31787 165.6857 28.36487 19.10689 81.15856 85.08208 71.92 160.5766 476.5021 78.21612 80.52814 208.1697 170.9468 148.6169 96.92955 127.4553 63.99372 323.7152 144.5998 283.0476 75.27428 145.644 42.07792 46.7245 122.8102 123.2688 131.1732 135.5121 60.39772 112.28 209.6676 104.6342 410.3269 148.7036 160.3243 127.5791 153.1571 136.9667 33.8717 201.5956 87.60148 230.7294 179.304 116.5572 71.8783 196.1032 244.5776 326.108 83.54707 65.63253 179.1287 117.7912 100.5578 182.4198 71.09133 127.1641 184.7397 764.5174 750.1892 1008.527 1004.373 249.2076 1011.885 1061.902 837.9646 230.9002 808.1162 528.4253 889.8957

Hub gene for module blue : MT-CO3

Module: brown

Number of genes in module: 527

Connectivity length: 527

Connectivity names: VAMP3 AGTRAP MTHFR EFHD2 CASP9 SPEN RCC2 CDA UBXN11 IFI6 SESN2 MARCKSL1 AGO4 AGO1 STK40 LSM10 PABPC4 PPT1 PLK3 AKR1A1 IFI44L GNG5 EVI5 BCAR3 DPYD TMEM167B C1orf162 ATP1A1 CD58 NBPF14 MCL1 CTSS S100A11 YY1AP1 MEF2D ARHGEF11 ETV3 CD1D PRDX6 RC3H1 ABL2 XPR1 IER5 ARPC5 PTGS2 PLA2G4A MIR181A1HG RNPEP MAPKAPK2 G0S2 ATF3 RPS6KC1 WDR26 LYST NLRP3 ID2 ADAM17 HPCAL1 FAM49A LAPTM4A RHOB CLIP4 LBH FEZ2 QPCT CDC42EP3 RTN4 VRK2 AFTPH RAB1A MOB1A CAPG TMEM127 RNF149 SLC20A1 PTPN18 PLEKHB2 KYNU TNFAIP6 ARL5A STAM2 BAZ2B GALNT3 UBR3 STAT1 BMPR2 RAPH1 ZDBF2 PNKD TUBA4A ACSL3 PID1 TRIP12 CAB39 UBE2F IRAK2 SH3BP5 AZI2 CRTAP MYD88 CTNNB1 ZDHHC3 UQCRC1 TKT PXK ALCAM ATG3 CD86 PARP9 GK5 DIPK2A RNF13 TSC22D2 SKIL FNDC3B OPA1 ACAP2 PCYT1A KIAA0232 SH3TC1 SLC2A9 WDR1 LAP3 TLR1 TMEM165 CXCL8 NAAA SCARB2 ANTXR2 RASGEF1B HERC3 TET2 PAPSS1 PLA2G12A SEC24D ANXA5 ELF2 FBXW7 TMEM154 DDX60L AC084871.1 LPCAT1 POLK ARSB VCAN ARRDC3 ST8SIA4 PGGT1B FEM1C AC116366.1 SEC24A SIL1 HBEGF LARP1 LCP2 STK10 ATP6V0E1 CPEB4 UIMC1 PRELID1 DOK3 CANX SERPINB1 SERPINB9 GCNT2 NEDD9 TBC1D7 AL138720.1 JARID2 KIF13A TRIM38 HIST1H2BJ LST1 FKBP5 MAPK14 KCTD20 CDKN1A TFEB PTCRA CNPY3 OGFRL1 SLC17A5 UBE2J1 SNX3 FOXO3 DSE CALHM6 MAN1A1 SMPDL3A SGK1 SASH1 SOD2 IGF2R QKI TNRC18 CYTH3 RAC1 AHR FAM126A SNX10 ZNRF2 AVL9 BLVRA UPP1 GUSB ASL TPST1 GTF2I HIP1 BRI3 PIK3CG PRKAR2B ATP6V1F TBXAS1 CUL1 TMEM176B SMARCD3 RHEB MSRA MSR1 BIN3 DOCK5 KIF13B ZNF703 RAB11FIP1 GPAT4 YTHDF3 NCOA2 UBE2W LY96 RIPK2 AZIN1-AS1 EXT1 SNTB1 FAM91A1 ST3GAL1 SLC45A4 DOCK8 MOB3B FAM214B RUSC2 ZFAND5 GNAQ AGTPBP1 ISCA1 CTSL NANS PTBP3 PBX3 ZBTB43 NIBAN2 ENG LCN2 NUP214 RAPGEF1 GFI1B CAMSAP1 AGPAT2 LARP4B WDR37 GDI2 SFMBT2 OPTN RSU1 MSRB2 OTUD1 CSGALNACT2 RASSF4 ALOX5 IPMK VSIR PSAP VCL PPIF ZCCHC24 PAPSS2 PTEN LIPA CPEB3 MYOF PI4K2A ARMH3 SLK CACUL1 WDR11 PTPRE ADAM8 RNH1 IRF7 PHLDA2 RHOG ILK NRIP3 DENND5A AMPD3 MTRNR2L8 FAR1 TSG101 HIPK3 COMMD9 AC090559.1 RTN3 FERMT3 NEAT1 KDM2A GRK2 ANKRD13D CHKA LAMTOR1 ATG16L2 RELT PAK1 GAB2 AP003086.1 PRCP SMCO4 ZC3H12C SIK2 PAFAH1B2 CBL APLP2 ADIPOR2 CD4 PTPN6 C3AR1 ETV6 H2AFJ LRMP VDR KMT2D TUBA1B DAZAP2 ACVR1B BLOC1S1 TBK1 RASSF3 LINC02384 GLIPR1 ATP2B1-AS1 FGD6 CKAP4 TCP11L2 SSH1 MED13L MLXIP CLIP1 SBNO1 GLT1D1 PARP4 FRY EPSTI1 RCBTB2 KPNA3 DNAJC3 STK24 IRS2 CARS2 TFDP1 ARHGEF40 OXA1L SLC7A7 STRN3 LGALS3 PELI2 PPM1A VTI1B ACTN1 PSEN1 ELMSAN1 SPTLC2 ATXN3 LGMN DICER1 SETD3 RCOR1 MARK3 AC127502.2 SLC12A6 SPG11 BLOC1S6 SQOR MAPK6 MYO5A ADAM10 PLEKHO2 SPG21 MAP2K1 CTSH EFL1 AP3S2 ZNF710 LRRK1 ATP6V0C PDPK1 CPPED1 APOBR AC009093.2 PPP4C ATP6V0D1 WWP2 CDYL2 MPHOSPH6 COTL1 ZDHHC7 MAP1LC3B CRK P2RX1 MINK1 RNASEK GABARAP PIK3R5 AKAP10 KSR1 LGALS9 TAOK1 ADAP2 NF1 EVI2B CCL3 CCL3L1 LASP1 GRN ABI3 FAM117A LRRC59 SPAG9 SUPT4H1 CLTC USP32 PECAM1 TTYH2 GRB2 SMIM5 SEC14L1 SLC38A10 SLC16A3 MBD2 SLC66A2 ARID3A GPX4 MIDN DOT1L OAZ1 ZBTB7A SH3GL1 TICAM1 VAV1 INSR PNPLA6 TRAPPC5 PRAM1 AC011511.2 ICAM1 CDKN2D TMEM205 MAN2B1 AC020916.1 PRKACA PKN1 AKAP8L TPM4 RAB8A MYO9B BST2 GMIP USF2 TYROBP CAPNS1 KCNK6 POU2F2 NECTIN2 VASP NAPA FTL FCGRT NLRP12 MYADM MBOAT7 LILRA6 LILRA2 LILRA1 C20orf194 C20orf27 PRNP GPCPD1 CST3 ABHD12 NCOA6 NORAD TOP1 B4GALT5 SS18L1 RGS19 NRIP1 APP MAP3K7CL IFNAR2 PFKL BID MAPK1 CHCHD10 GRK3 MN1 YWHAH TOM1 LGALS2 GGA1 SH3BP1 LGALS1 ATF4 EP300 NAGA PACSIN2 TSPO SCO2 PLCXD1 CSF2RA IL3RA DHRSX CFP AL034397.3 DIAPH2 BEX3 IL13RA1 SASH3 ATP6AP1 G6PD

Connectivity values: 33.81632 24.66624 20.0198 105.1054 24.31486 93.6351 42.15493 14.69751 14.9267 36.85652 16.99697 47.41372 47.08337 34.1264 48.05687 35.82565 78.63077 36.83145 62.7469 36.09263 22.81666 122.3388 52.36659 11.15896 278.6042 45.21795 51.96356 110.6533 77.83215 15.18 172.1614 160.3494 140.0419 98.0137 74.96569 7.211168 47.81917 5.250224 120.6977 170.3318 47.40178 110.268 100.4382 124.281 4.149031 5.740823 118.5753 25.33418 117.5326 24.38912 37.48406 57.06731 190.0091 141.5064 27.408 108.0005 115.5536 85.89718 74.84549 76.6773 21.36717 53.3408 81.08068 28.92573 5.146733 60.04699 173.1343 50.20974 104.1021 202.8385 133.8577 24.4939 28.4334 239.0399 47.57504 34.91327 116.6141 40.41167 3.644586 66.9492 40.56816 98.16949 20.81624 85.53193 110.2838 62.76711 16.8076 57.94834 45.59353 103.8256 92.8178 37.23848 176.9987 118.2367 67.75674 48.74884 57.39539 42.50705 18.88478 19.79774 173.0768 43.55076 36.84289 129.2962 48.28656 47.53255 68.82116 26.16261 40.16939 20.17621 24.42595 88.93202 98.274 163.5306 166.68 130.1522 205.5274 40.16523 98.74224 23.03249 5.363429 67.03748 60.16822 19.1739 78.932 27.05729 40.17348 35.4564 62.408 74.81494 69.62168 152.39 20.13563 19.73302 50.84692 75.49819 199.9678 198.3377 42.8625 43.15653 9.735712 34.9122 48.64702 30.08781 51.49925 52.41991 76.69921 51.1752 33.91479 12.16484 62.9899 34.65721 14.01185 70.52216 179.477 194.4619 136.506 77.36242 105.7764 106.5289 13.32537 92.95678 95.27051 108.4566 15.58117 133.8558 16.51835 8.956449 241.1913 19.62061 84.24538 10.50023 85.61752 205.3864 90.0796 86.14937 41.93911 30.23617 11.31096 61.66885 41.9829 41.88724 82.88462 126.6904 132.7422 57.9696 28.04236 86.07375 5.084645 61.83203 9.520422 182.2637 98.86086 234.7806 44.66562 32.01332 158.8557 129.4746 35.30292 44.74982 104.0659 89.09067 27.52394 118.325 41.32356 18.19997 28.44072 113.1155 39.61755 142.0435 48.59896 21.47362 100.5794 56.97594 89.57636 14.59006 5.427317 115.0397 45.294 13.20731 63.02541 44.01167 87.49825 6.80879 156.034 39.12394 132.0967 175.1901 71.80152 19.17956 48.82133 17.70414 77.88587 56.53243 69.68783 105.3731 39.9385 247.8442 16.12031 14.59597 7.89263 132.6447 140.9876 196.8209 126.5382 15.12003 46.92074 137.4157 67.61213 79.09569 8.934144 17.46408 4.706754 121.4508 253.338 7.345157 24.03858 11.49435 104.7596 87.2701 91.91103 136.6204 139.0395 63.30914 10.15586 28.22671 191.9585 7.289793 33.87722 51.22009 75.10411 121.4668 52.58919 37.66517 4.140389 8.341132 152.7265 27.91291 33.06676 7.560796 43.8597 86.25457 52.71896 69.71853 42.52773 96.5188 21.14109 67.86464 25.99182 6.648659 90.16683 34.58751 8.192805 107.0123 20.19922 91.47358 116.4308 86.85908 71.07439 37.80361 14.67052 72.78258 51.29699 355.9122 233.4068 68.42157 48.70774 88.46836 73.02788 33.39585 44.09734 54.85253 135.0334 65.372 33.26485 7.101381 7.934428 40.78917 60.47461 83.03596 86.32403 70.42878 45.81873 45.13096 8.145013 176.86 79.11802 93.76056 15.16759 32.26951 107.2603 172.6243 22.65767 80.44954 66.16052 138.8083 13.08257 59.50102 58.49744 11.95456 21.10426 99.3678 39.63984 307.2842 69.51688 101.264 82.3177 18.55134 89.24296 53.94316 90.46702 19.87917 94.17779 139.1326 156.3088 96.57398 37.32394 37.7475 10.5371 42.46185 20.30355 88.2321 63.10742 106.229 113.5483 63.56195 41.64981 128.5101 54.32593 71.90696 72.54067 11.53538 88.31832 37.4956 146.3648 179.813 32.3801 105.0696 65.56755 81.67664 30.55781 85.77951 88.5659 122.8693 16.72326 30.0226 127.1141 31.01843 28.0272 48.57666 32.61841 13.24198 209.9734 37.32642 30.07049 9.942227 36.43011 55.00378 73.75429 122.7443 24.30257 23.55603 181.2923 49.61408 178.5226 59.19787 26.31033 42.09775 210.1032 191.7698 239.3734 66.14655 45.2081 29.08844 86.32877 12.01498 98.91497 117.9041 18.39588 9.229013 47.6258 34.64468 13.7379 59.4615 33.79231 180.4728 68.1424 88.31884 67.27736 55.30728 9.338562 194.8365 9.897293 140.0306 28.59103 61.84817 102.1336 58.07598 25.94284 169.6253 70.90151 40.72957 333.8292 90.14568 45.97051 26.47786 80.25917 21.18611 20.10422 97.5442 19.00217 13.44218 40.96914 121.1536 17.25841 56.89615 80.48443 25.89542 72.97761 92.75289 107.2344 66.01524 181.7316 52.79484 37.15493 58.32523 88.0548 58.89648 11.78499 69.06349 7.792178 73.30412 77.59174 619.0914 40.77751 4.70606 73.14929 17.83304 4.585526 9.033389 8.764037 32.80685 17.76803 62.23716 259.9415 74.09751 44.58273 60.93444 91.0942 266.8687 79.56793 14.61626 39.68314 142.0077 46.71677 19.25541 98.64708 26.74802 90.29979 148.1713 56.1387 66.21784 3.62093 53.90369 74.85015 10.80192 44.66367 27.35036 105.8545 140.3302 159.8314 6.86365 116.4613 93.35973 23.82677 7.210708 20.96608 29.73847 60.35716 23.39888 7.027871 127.795 27.43684 14.13535 47.44367 21.88839 13.90162

Hub gene for module brown : FTL

Module: black

Number of genes in module: 77

Connectivity length: 77

Connectivity names: RERE AL078459.1 CEPT1 LY9 RCSD1 RHEX TRAF5 ZNF669 ADCY3 RASGRP3 LINC02245 EIF2AK3 IGKC AFF3 MIR4435-2HG SP140 APPL1 BTLA ST6GAL1 NSUN7 JCHAIN ARHGAP24 BANK1 MTMR12 ZCCHC10 EBF1 SIMC1 AL365272.1 CD83 BTN2A2 CD24 PTPRK AL589693.1 TAGAP IGF2BP3 AUTS2 AC073111.4 BLK KHDRBS3 CCDC6 LINC01374 ENTPD1-AS1 BLNK INPP5A HPS5 ATF7IP RNF41 SLC41A2 USP12 RUBCNL RB1 SETDB2 IGHM TMEM62 SECISBP2L GABPB1 MAPK8IP3 ACSM1 RABEP2 CD19 PLCG2 P2RX5 ATP2A3 NUP88 CABLES1 TCF4 TCF3 AC119396.1 ZNF737 PRKD2 CD37 MYBPC2 SLC37A1 ICOSLG IGLC3 RPGR SLC9A7

Connectivity values: 141.6715 12.27025 45.21288 45.27458 94.32884 4.655732 39.97742 16.92039 17.63668 24.98735 19.01727 127.4301 48.78053 110.1279 65.44226 96.60462 58.66573 17.23643 100.4929 5.901031 5.785354 48.35323 46.65893 53.53658 95.76346 40.44532 54.07149 10.23219 165.3295 16.37601 4.409117 25.85591 33.15501 85.51597 65.95123 127.1828 13.97603 39.52315 4.806686 75.57738 4.927405 34.08103 5.743783 97.57262 31.95944 179.3975 24.31712 8.907031 133.1057 19.60291 119.419 23.08127 46.52017 29.80458 79.48845 129.378 69.0045 4.351491 31.27914 4.193354 144.9952 21.34215 58.00485 111.2571 5.687373 43.86219 30.42386 51.16319 26.84624 42.63126 235.0358 3.220114 34.10288 11.96645 14.10784 46.65798 21.73572

Hub gene for module black : CD37

Module: red

Number of genes in module: 88

Connectivity length: 88

Connectivity names: AL021155.5 CROCC IFFO2 AL360012.1 ZC3H12A PTCH2 NASP AC103591.3 AC239799.2 BTG2 PPP1R15B EFCAB2 SRSF7 AC012447.1 NR4A2 GLS CSRNP1 KIF9 ARF4 SEC62 NOCT NR3C2 RAPGEF2 HMGCS1 FAM53C EGR1 GALNT10 AC022217.3 AL121944.1 TRIM39 GNL1 PPP1R10 TNF BRD2 AMD1 RABGEF1 MEPCE B4GALT1 AL499604.1 STX17-AS1 NR4A3 RALGDS TUBB4B ARL5B SIRT1 RRP12 WDR74 PCF11 SLC38A2 NR4A1 AL355075.4 AL627171.2 LINC01588 GCH1 AL691403.1 EIF5 TP53BP1 COQ7 EEF2K ATXN2L POLR2A TMEM107 NDEL1 AC007952.4 NUFIP2 CASC3 CCDC200 LINC00910 TEX14 EIF4A3 WDR45B CHMP1B GADD45B PEX11G LDLR HOOK2 DDX39A IQCN ELL ERCC1 FOSB PPP1R15A UBE2S ZNF335 PDXK GTPBP1 Z93241.1 ZFY

Connectivity values: 48.09208 106.4031 48.69178 24.05504 36.05282 17.20236 118.5715 74.12725 14.65671 158.0959 68.02777 48.26106 239.096 44.16072 124.0713 193.1598 61.65765 22.39564 127.7594 247.9263 52.95247 26.75582 102.4354 50.23686 46.02438 29.49131 54.9237 96.82073 22.24502 16.27782 80.48263 108.2385 39.42226 106.8712 130.1267 163.8102 26.62819 149.3978 57.06464 18.94019 46.46925 63.23653 83.87403 42.27033 71.76515 56.9167 72.76241 83.77331 185.3577 34.60679 19.17849 50.21651 35.08521 91.07397 51.47529 273.2813 56.232 26.69864 26.29923 102.6354 133.1264 36.41234 110.6017 70.99539 160.6074 57.58353 27.35762 60.62338 108.6839 77.43463 65.98056 74.38962 114.6985 14.34274 55.56685 46.8871 88.26503 24.02084 66.02526 65.16029 109.2941 212.0981 68.81653 71.07092 58.32862 69.80772 34.05572 40.51104

Hub gene for module red : EIF5

Module: pink

Number of genes in module: 52

Connectivity length: 52

Connectivity names: PLEKHM2 LAPTM5 ST6GALNAC3 CD55 KMO PRKCE MAP4K4 CCNYL1 PIKFYVE IQSEC1 UBE2E2 TRAK1 SLC49A4 HES1 BMP2K DAPP1 GAB1 GAPT MEF2C SNX2 CXXC5 LY86 HLA-DPA1 TNFRSF21 STX7 JAZF1 VPS41 LAT2 NCF1 GSAP HMBOX1 MTSS1 PTK2 SAMD8 DNMBP DNMBP-AS1 TRIM44 BAZ2A WASHC4 SH2B3 WDFY2 SIPA1L1 USP8 CLCN7 PHKB HERPUD1 ATPAF2 RBBP8 BRD4 SPINT2 PARVB TBC1D22A

Connectivity values: 86.61179 291.675 33.04699 227.4628 7.893713 116.1817 203.6673 31.25621 65.26148 118.6259 118.2168 78.40672 66.57368 14.76222 49.57282 43.25692 23.89553 8.465435 47.75713 104.9778 21.87103 17.58417 82.27329 8.802882 44.00095 98.82248 55.01053 27.65005 60.17991 32.00091 68.49977 84.40135 76.39287 92.43033 55.42762 9.725622 74.00734 87.90248 97.50489 103.0938 108.6827 305.0989 128.4117 25.15094 79.04278 121.7764 22.49256 22.86923 188.3557 34.17816 32.56763 157.4779

Hub gene for module pink : SIPA1L1

Module: yellow

Number of genes in module: 175

Connectivity length: 175

Connectivity names: SH3BGRL3 AGO3 PRDX1 EFCAB14 GNAI3 CD53 RAP1A GDAP2 S100A10 S100A6 S100A4 TPM3 TAGLN2 ARHGAP30 ARF1 IRF2BP2 YWHAQ NCOA1 HADHA SNX17 NRBP1 YIPF4 ACTR2 PCBP1 VAMP5 CYTOR PLEKHA3 CFLAR ARPC2 CTDSP1 EIF4E2 LRRFIP1 ARPC4 BRK1 PDCD6IP RHOA EIF4E3 CHMP2B COX17 HCLS1 SEC61A1 RPN1 ATP1B3 SLC9A9 SSR3 TIPARP NDUFB5 LPP C4orf48 GRPEL1 PPP3CA C4orf3 SCLT1 SPCS3 GLRX ETF1 DIAPH1 NR3C1 SSR1 MAP3K5 RNASET2 ACTB KDELR2 TAX1BP1 DBNL OGDH ARPC1B LAMTOR4 TSC22D4 ZNHIT1 TPK1 ERICH1 DOK2 ENY2 TMEM65 AC131568.1 LY6E CYC1 VCP CLTA KLF9 ANXA1 PRXL2C ERP44 TXN HSPA5 VIM SRGN GHITM PGAM1 IFITM2 POLR2L TSPAN32 TRIM22 TAF10 AHNAK COX8A POLD4 PPP1CA RAB6A UCP2 CARD16 ZBTB16 SORL1 GAPDH MLF2 TPI1 GXYLT1 TMBIM6 PRR13 MYL6 ATP5F1B TMBIM4 CHST11 ARPC3 PXN CHFR CPB2-AS1 LCP1 ITM2B SCFD1 HECTD1 PCNX1 GALC IFI27L2 PDIA3 SERF2 CASC4 COX5A UBE2Q2 PSTPIP1 SEC11A SLCO3A1 PGP ELOB UBN1 BFAR MT2A FAM192A CYBA ZZEF1 XAF1 ACADVL EIF4A1 PSMB3 IFI35 SNF8 DCAF7 ERN1 JPT1 ARHGDIA ANAPC11 UQCR11 MYDGF WDR83OS STX10 PGLS ACTN4 EMP3 KDELR1 UBE2M RPN2 RTF2 ATP5F1E MX1 ITGB2 TRABD SMS UBA1 PLP2 MSN PGK1 VMA21 IDH3G ARHGAP4

Connectivity values: 356.5608 124.509 68.54464 60.0093 122.7779 218.3364 241.9823 38.25415 244.1684 276.8131 293.0878 284.1741 210.8293 78.94798 197.5074 135.0392 129.6748 165.8142 84.79893 34.46782 62.5226 86.27149 243.5992 148.651 50.18622 66.04872 49.91271 181.7974 336.9104 40.58854 64.76794 308.5602 110.3524 93.30525 105.9322 253.9693 50.36074 71.27095 65.61104 116.0048 46.47921 57.81149 159.7181 73.90427 60.71466 77.42055 44.69845 189.2279 48.74745 51.54985 197.5841 86.60067 132.2343 117.9487 63.83729 145.1304 154.8111 244.791 45.98035 106.0432 139.136 527.736 35.60885 249.9139 51.6297 98.57082 206.7446 122.7007 34.75208 65.20034 51.57173 81.75755 59.9539 105.2455 67.42643 39.68403 110.1612 35.82539 99.41194 117.4297 79.79817 189.4253 24.95631 106.3967 93.31612 199.2565 377.4457 391.6202 97.60598 54.05801 197.5102 89.03813 22.39405 87.39868 85.68651 147.0596 125.5563 63.00846 82.06403 88.14549 108.6832 55.31376 99.10127 160.7927 315.1561 44.78826 84.36362 35.86588 185.4741 93.71683 389.921 117.5607 87.98018 287.556 301.8915 62.6328 74.74676 42.8787 211.5667 342.9435 171.1078 141.5752 178.8057 24.18236 33.09698 203.5557 402.4845 61.11528 99.49214 78.79561 27.55277 132.0414 165.8986 33.9684 206.3053 79.11304 53.01022 124.2368 50.20173 261.6958 108.2599 48.83463 31.82378 260.0099 89.61971 23.21973 60.91446 55.23107 97.3038 55.27783 110.1911 54.37995 181.1844 81.54529 104.7448 29.98765 84.84372 65.23879 286.2452 39.77121 51.03867 81.14936 91.50245 467.7366 59.09725 115.1844 63.87354 48.28919 28.09376 60.25441 220.4963 178.2568 36.80871 39.06881 69.28484

Hub gene for module yellow : ACTB

**Supplementary References:**

1. Butler A, Hoffman P, Smibert P, Papalexi E, Satija R. Integrating single-cell transcriptomic data across different conditions, technologies, and species. *Nature biotechnology.* 2018;36(5):411-420.

2. Satija R, Farrell JA, Gennert D, Schier AF, Regev A. Spatial reconstruction of single-cell gene expression data. *Nature Biotechnology.* 2015;33(5):495-502.

3. Qu, H.-Q., Ostberg, K., Slater, D. J., Wang, F., Snyder, J., Hou, C., Connolly, J. J., March, M. E., Glessner, J. T., Kao, C., & Hakonarson, H. Sex-Specific Differences in Peripheral Blood Mononuclear Cells Revealed by Single-Cell Transcriptome Analysis. Submitted.

4. Aran D, Looney AP, Liu L, et al. Reference-based analysis of lung single-cell sequencing reveals a transitional profibrotic macrophage. *Nature immunology.* 2019;20(2):163-172.

5. Kramer O, Kramer O. Scikit-learn. *Machine learning for evolution strategies.* 2016:45-53.

6. Zhang B, Horvath S. A general framework for weighted gene co-expression network analysis. *Statistical applications in genetics and molecular biology.* 2005;4(1).

7. Langfelder P, Horvath S. WGCNA: an R package for weighted correlation network analysis. *BMC bioinformatics.* 2008;9:1-13.

8. Yu G, Wang L-G, Han Y, He Q-Y. clusterProfiler: an R package for comparing biological themes among gene clusters. *Omics: a journal of integrative biology.* 2012;16(5):284-287.

9. Dolgalev I. msigdbr: MSigDB gene sets for multiple organisms in a tidy data format. *R package version.* 2020;7(1).

10. Pennell LM, Galligan CL, Fish EN. Sex affects immunity. *Journal of autoimmunity.* 2012;38(2-3):J282-J291.

11. Kleiveland CR. Peripheral blood mononuclear cells. *The Impact of Food Bioactives on Health: in vitro and ex vivo models.* 2015:161-167.

12. Webb DC, Cai Y, Matthaei KI, Foster PS. Comparative roles of IL-4, IL-13, and IL-4Rα in dendritic cell maturation and CD4+ Th2 cell function. *The Journal of Immunology.* 2007;178(1):219-227.

13. Paul MS, Ohashi PS. The roles of CD8+ T cell subsets in antitumor immunity. *Trends in cell biology.* 2020;30(9):695-704.

14. Bilate AM, Lafaille JJ. Induced CD4+ Foxp3+ regulatory T cells in immune tolerance. *Annual review of immunology.* 2012;30(1):733-758.

15. Tsanaktsi A, Solomou EE, Liossis S-NC. Th1/17 cells, a subset of Th17 cells, are expanded in patients with active systemic lupus erythematosus. *Clinical Immunology.* 2018;195:101-106.

16. Dimitrijević M, Arsenović-Ranin N, Bufan B, et al. Collagen-induced arthritis in Dark Agouti rats as a model for study of immunological sexual dimorphisms in the human disease. *Experimental and Molecular Pathology.* 2018;105(1):10-22.

17. Dodd KC, Menon M. Sex bias in lymphocytes: Implications for autoimmune diseases. *Frontiers in Immunology.* 2022;13:945762.

18. Kwon H, Schafer JM, Song N-J, et al. Androgen conspires with the CD8+ T cell exhaustion program and contributes to sex bias in cancer. *Science immunology.* 2022;7(73):eabq2630.

19. Dimitrijević M, Arsenović-Ranin N, Kosec D, et al. Sex differences in Tfh cell help to B cells contribute to sexual dimorphism in severity of rat collagen-induced arthritis. *Scientific reports.* 2020;10(1):1214.

20. Greenlees R, Mihelec M, Yousoof S, et al. Mutations in SIPA1L3 cause eye defects through disruption of cell polarity and cytoskeleton organization. *Human molecular genetics.* 2015;24(20):5789-5804.

21. Bécart S, Altman A. SWAP‐70‐like adapter of T cells: a novel Lck‐regulated guanine nucleotide exchange factor coordinating actin cytoskeleton reorganization and Ca2+ signaling in T cells. *Immunological reviews.* 2009;232(1):319-333.

22. Kardos GR, Dai MS, Robertson GP. Growth inhibitory effects of large subunit ribosomal proteins in melanoma. *Pigment cell & melanoma research.* 2014;27(5):801-812.

23. Araki K, Morita M, Bederman AG, et al. Translation is actively regulated during the differentiation of CD8+ effector T cells. *Nature immunology.* 2017;18(9):1046-1057.

24. Hamilton SE, Jameson SC. CD8 T cell quiescence revisited. *Trends in immunology.* 2012;33(5):224-230.

25. Bretones G, Delgado MD, León J. Myc and cell cycle control. *Biochimica et Biophysica Acta (BBA)-Gene Regulatory Mechanisms.* 2015;1849(5):506-516.

26. Guo A, Huang H, Zhu Z, et al. cBAF complex components and MYC cooperate early in CD8+ T cell fate. *Nature.* 2022;607(7917):135-141.

27. Wang R, Dillon CP, Shi LZ, et al. The transcription factor Myc controls metabolic reprogramming upon T lymphocyte activation. *Immunity.* 2011;35(6):871-882.

28. Papa S, Martino PL, Capitanio G, et al. The oxidative phosphorylation system in mammalian mitochondria. *Advances in Mitochondrial Medicine.* 2012:3-37.

29. Vardhana SA, Hwee MA, Berisa M, et al. Impaired mitochondrial oxidative phosphorylation limits the self-renewal of T cells exposed to persistent antigen. *Nature immunology.* 2020;21(9):1022-1033.

30. Mjösberg J, Svensson J, Johansson E, et al. Systemic reduction of functionally suppressive CD4dimCD25highFoxp3+ Tregs in human second trimester pregnancy is induced by progesterone and 17β-estradiol. *The Journal of Immunology.* 2009;183(1):759-769.

31. Paulin FE, Campbell LE, O'Brien K, Loughlin J, Proud CG. Eukaryotic translation initiation factor 5 (eIF5) acts as a classical GTPase-activator protein. *Current Biology.* 2001;11(1):55-59.

32. Abdul Hamid S. *Investigating the role of PIP4K in Immune System Regulation and P53-Inactivated Cancers*, University of Southampton; 2021.

33. Hall BM. CD4+ CD25+ T regulatory cells in transplantation tolerance: 25 years on. *Transplantation.* 2016;100(12):2533-2547.

34. Akbar AN, Lord JM, Salmon M. IFN-α and IFN-β: a link between immune memory and chronic inflammation. *Immunology today.* 2000;21(7):337-342.

35. Golding A, Rosen A, Petri M, Akhter E, Andrade F. Interferon‐alpha regulates the dynamic balance between human activated regulatory and effector T cells: implications for antiviral and autoimmune responses. *Immunology.* 2010;131(1):107-117.

36. Zhen Y, Zheng J, Zhao Y. Regulatory CD4+ CD25+ T cells and macrophages: communication between two regulators of effector T cells. *Inflammation Research.* 2008;57:564-570.

37. Rs J. The immune microenvironment in human papilloma virus-induced cervical lesions—Evidence for estrogen as an immunomodulator. *Frontiers in Cellular and Infection Microbiology.* 2021;11:649815.
